# Patellar Dislocation: Workup and Decision-Making

**DOI:** 10.7759/cureus.46743

**Published:** 2023-10-09

**Authors:** Panagiotis V Samelis, Panagiotis Koulouvaris, Olga Savvidou, Andreas Mavrogenis, Vasileios P Samelis, Panayiotis J Papagelopoulos

**Affiliations:** 1 Orthopaedics, Children’s General Hospital Panagiotis and Aglaia Kyriakou, Athens, GRC; 2 Orthopaedics, Attikon University Hospital, Athens, GRC; 3 Orthopaedic Surgery, National and Kapodistrian University of Athens School of Medicine, Athens, GRC; 4 Orthopaedics, National and Kapodistrian University of Athens School of Medicine, Athens, GRC; 5 Medicine, European University Cyprus, Nicosia, CYP

**Keywords:** patella, dislocation, mpfl, mptl, mpml, extensor, mechanism, knee, vmo, valgus

## Abstract

Acute patellar dislocation (PD) is usually a problem of adolescents and young adults. In most cases, it is a sports-related injury. It is the result of an indirect force on the knee joint, which leads to valgus and external rotation of the tibia relative to the femur. PD is unlikely to occur on a knee with normal patellofemoral joint (PFJ) anatomy. Acute PD consists of an acute injury of the ligamentous medial patellar stabilizers in the background of factors predisposing to patellar instability. These factors are classified into three groups. The first group refers to the integrity of the ligamentous medial patellar restraints, particularly, the medial patellofemoral ligament (MPFL). The second group refers to an abnormal PFJ anatomy, which renders the patella inherently unstable inside the trochlea. The third group refers to the overall axial and torsional profile of the lower limb and to systemic factors, such as ligament laxity and neuromuscular coordination of movement. PD at a younger age is associated with an increased number and severity of patellar instability predisposing factors and lower stress to dislocate the patella. Acute primary PD is usually treated conservatively, while surgical treatment is reserved for recurrent PD. The aim of treatment is to restore the stability and function of the PFJ and to reduce the risk of patellar redislocation. Surgical procedures to treat patellar instability are classified into non-anatomic and anatomic procedures. Non-anatomic procedures are extensor mechanism realignment techniques that aim to center the patella into the trochlear groove. Anatomic procedures aim to restore the PFJ anatomy (ruptured ligaments, osteochondral fractures), which has been severed after the first incident of PD. Anatomic procedures, especially MPFL reconstruction, are more effective in preventing recurrent PD, compared with non-anatomic techniques. Theoretically, all factors that affect PFJ stability should be evaluated and, if possible, addressed. This is practically impossible. Considering that the MPFL ruptures in almost all PDs, MPFL reconstruction is the primary procedure, which is currently selected by most surgeons as a first-line treatment for patients with recurrent PD. Restoration of the axial and torsional alignment of the lower limbs is also increasingly implemented by surgeons. Non-anatomic surgical techniques, such as tibial-tuberosity osteotomy, are used as an adjunct to anatomic procedures. In the presence of multiple PFJ instability factors, acute MPFL reconstruction may be the treatment of choice for acute primary PD as well. Skeletal immaturity of the patient precludes osseous procedures to avoid premature physis closure and subsequent limb deformity. Unfortunately, restoration of the patient's previous activity level or participation in more strenuous sports is questionable and not easy to predict. In the case of competitive athletes, PD may prevent participation in elite levels of sports.

## Introduction and background

The patella is the largest sesamoid bone in the body. The articular surface of the patella is divided by a vertical median ridge to a larger lateral facet and a medial facet. Medially to the medial facet, a secondary vertical ridge separates the medial facet from a third facet, called the odd facet. The articular cartilage of the patella is the thickest of the human body (up to 7 mm near the median ridge). The patella articulates with the trochlea to create the patellofemoral joint (PFJ) [1].

The trochlea is a longitudinal articular groove on the anterior part of the distal femoral epiphysis. It is formed between two articular walls or facets, the medial, and the lateral facet [1]. The lateral trochlear facet is higher (more prominent) and extends more proximally than the medial trochlear facet. The depth of the trochlea is approximately 5.2 mm when measured on MRI at 3cm above to the knee joint line [2]. Its distal part is deeper than the proximal part [2]. The thickness of the articular cartilage of the trochlea is 2-3 mm. The articular cartilage of the lateral trochlear facet is thicker than the cartilage of the medial trochlear facet [3].

The cartilaginous contours of the patella and the trochlea do not accurately reflect the respective contour of the subchondral bone [3]. In the normal PFJ, the patella is centered in the trochlear groove after 30⁰ of knee flexion [4]. The trochlea is the major osseous constraint against lateral patellar dislocation (PD) beyond 30⁰ of knee flexion [1,2,4,5].

The patella not only transmits the force of the quadriceps contraction to the tibia [6] but also increases the moment arm of the quadriceps as well [1]. From a biomechanical aspect, the joint contact forces of the PFJ may reach up to 20 times the body weight during sports [6]. To maintain this function, the patella is stabilized on the femoral trochlea by means of ligaments (static stabilizers), muscles (dynamic stabilizers), and bones (passive stabilizers) [7]. Depending on the amount and the rate of loading of the PFJ, a wide range of symptoms may emerge, from PFJ pain to PD [8].

**Figure 1:**
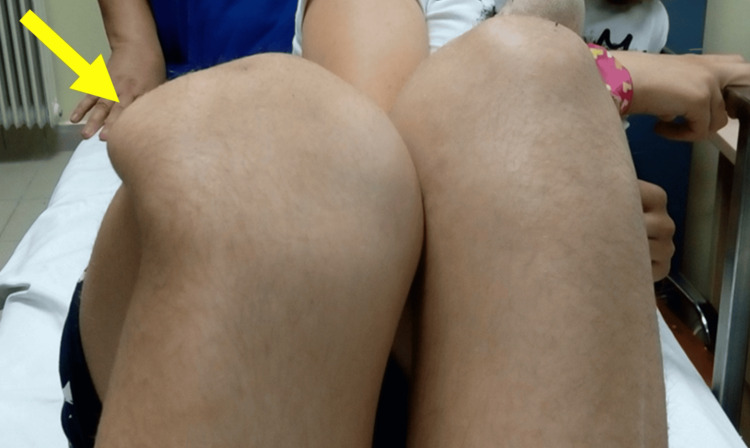
Acute, locked right PD in a 13-year-old volleyball player Arrow indicates the laterally dislocated patella. Figure created by Panagiotis Samelis.

Acute (primary, traumatic, first-time) PD usually affects younger ages and, in most cases, it is a sports-related injury (Figure 1) [9]. The majority of PDs are the result of an indirect (non-contact) valgus force on a moderately flexed knee. At the time of injury, the hip is in mild flexion, the quadriceps is contracted, the femur is internally rotated, the tibia is externally rotated, and the foot steps firm on the ground [10].

**Figure 2:**
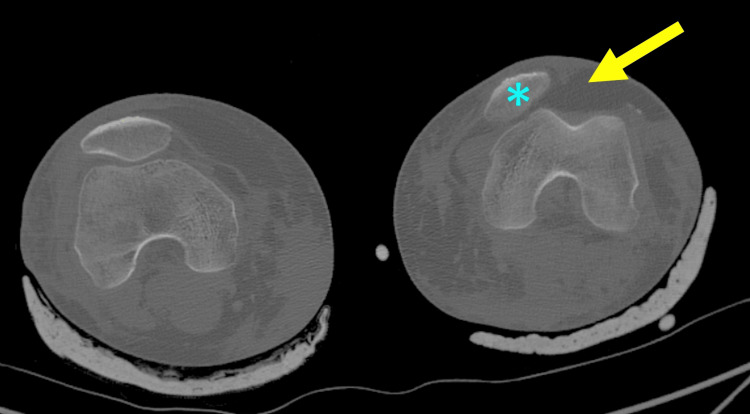
Medial dislocation of the left patella in a 50-year-old woman after a direct blow on the lateral surface of the left knee during a car accident Asterisk indicates the medially displaced left patella. Arrow indicates the direct blow on the lateral side of the patella. Figure created by Panagiotis Samelis.

PD after a medial or lateral direct blow on the patella is unlikely to occur and is usually observed in the context of severe trauma (Figure 2) [9].

The process of acute PD starts with an injury of the medial ligamentous stabilizers of the patella, especially the medial patellofemoral ligament (MPFL) [11,12]. Underlying abnormal PFJ anatomy, especially trochlear dysplasia (TD), as well as other factors that predispose to PFJ instability, are usually present in cases of PD and have to be evaluated prior to treatment selection [8,13]. A pathologic axial and torsional profile of the lower limb has to be assessed and addressed as well [14]. Systemic factors of the patient, such as generalized ligament laxity and poor neuromuscular coordination, may further exacerbate patellar instability and lead to PD or PFJ pain [15]. PD at a younger age is associated with increased number and severity of patellar instability predisposing factors and a lower threshold to dislocate the patella [16].

Acute primary PD is usually treated conservatively, while surgical treatment is reserved for recurrent PD [17]. The aim of treatment is to restore the stability and function of the PFJ and to reduce the risk of patellar redislocation [8]. Surgical techniques to treat patellar instability are classified into non-anatomic and anatomic procedures [14]. Non-anatomic procedures are extensor mechanism realignment techniques that aim to center the patella into the trochlear groove [14]. Anatomic procedures aim to restore the PFJ anatomy (ruptured ligaments, osteochondral fractures), which has been severed after the first incident of PD. Anatomic procedures, especially MPFL reconstruction, are more effective in preventing recurrent PD, compared with non-anatomic techniques [18]. Considering that the MPFL ruptures in almost all PDs, MPFL reconstruction is the primary procedure, which is widely selected by surgeons as a first-line treatment for most patients with recurrent PD [19]. Restoration of the axial and torsional alignment of the lower limbs is also increasingly applied by surgeons [14]. Non-anatomic surgical techniques, such as tibial-tuberosity osteotomy, are used in addition to anatomic procedures [20]. In the presence of PFJ instability factors, acute MPFL reconstruction may be the treatment of choice for acute primary PD as well [17]. Skeletal immaturity of the patient precludes osseous procedures to avoid premature physis closure and subsequent limb deformity [21]. Unfortunately, restoration of the patient’s previous activity level or participation in more strenuous sports is questionable and not easy to predict. In the case of competitive athletes, PD may lead to the end of a sports career [22,23].

**Figure 3:**
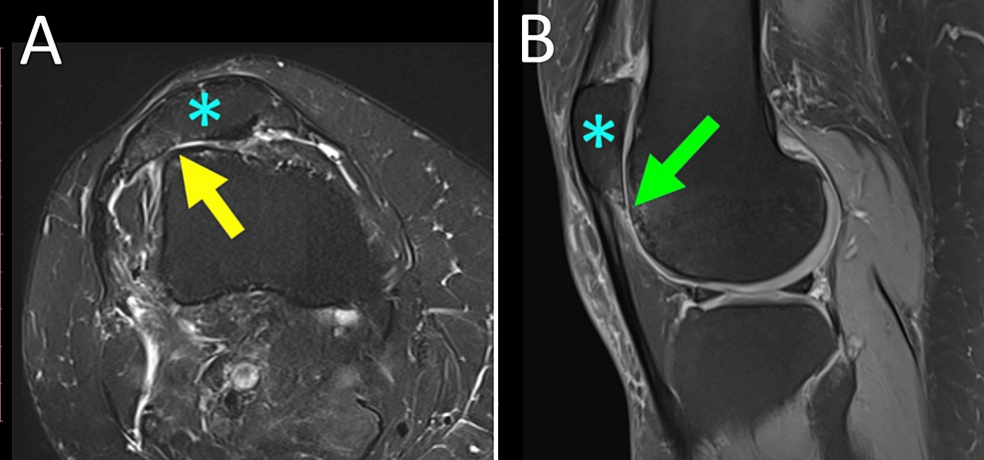
PFJ arthritis in a 48-year-old woman with chronic patellar instability and patella alta (A) Axial CT scan of the right knee. Lateral patellar subluxation (asterisk) with marked PFJ arthritis (yellow arrow) is evident. (B) Sagittal CT scan of the right knee. Patella alta (asterisk) and PFJ arthritis are evident (green arrow). Figure created by Panagiotis Samelis.

PD is still a riddle, in relation to its etiology, the risk of recurrence, and the selection of the appropriate treatment to obtain optimal long-term results for the patient, especially the athlete. In every patient, trauma, preexisting abnormal PFJ anatomy, and the overall neuromuscular status have to be evaluated to decide the proper treatment. Evaluation and treatment of acute (primary, first, traumatic) PD is crucial to prevent recurrent dislocation and to allow the patient to resume previous daily and athletic activities [8]. Untreated PFJ instability may avert the patient from athletic participation and lower the patient’s self-confidence. Furthermore, persisting patellar instability results in chronic abnormal PFJ loads and, besides pain and discomfort, leads to PFJ arthritis and the need for reconstruction surgery (Figure 3) [8,24,25].

## Review

PD affects younger ages

Acute traumatic (non-congenital, non-habitual) PD is predominantly a problem of adolescents and young adults.

Gravesen et al. conducted a retrospective epidemiologic study over a 20-year period in Denmark. The authors found that the mean incidence of acute PD was 42 per 100,000 person-years. The ratio of PD was similar between men (47%) and women (53%) [26]. Young females aged 10-17 had the highest incidence of acute PD (108 per 100,000 person-years) [26]. The same study showed that the risk of recurrent PD was 22.7%. Young girls, aged 10-17, had the highest risk of recurrent dislocation (36.8%). The overall risk of contralateral PD was 5.8%; however, in patients aged 10-17 years, this risk almost doubled (11.1%) [26].

In a two-year prospective study, Nietosvaara et al. found that in Finland the incidence of acute PD was 107:100,000 in the 9-15-year-old age group and 43:100,000 in children younger than 16 years. Two-thirds of patients were girls [27]. Fithian et al. calculated the incidence of primary PD for different age groups [28]. Thus, the incidence of primary PD was 29:100,000 in the age group 10-17 years, 9:100,000 in the age group 18-29 years, and only 1:100,000 in the age group older than 30 years [28]. In a selected population based on insurance status, Atkin et al. found an overall incidence of acute PD of 7:100,000 [9]. However, in the 10-19-year age group, the incidence of acute PD increased to 31:100,000 [9].

Whatever the type of the study or the population, on which it is based, all studies agree that acute traumatic PD affects mainly adolescents and young adults who are involved in some type of sports [9]. A possible explanation is that young people usually participate in sports in a competitive rather than a recreational manner. Furthermore, the inherently abnormal patellofemoral anatomy, which usually underlies PFJ instability, is expected to become symptomatic in younger patients, when gradual participation in higher levels of sports imposes higher loads on the PFJ. The combination of increased physical stress and abnormal anatomy are the key points of patellar instability in younger people [16]. Unfortunately, two-thirds of the patients have open physes, which limits the available treatment options solely to soft-tissue procedures [16].

Epidemiologic studies are not unanimous regarding gender as a risk factor for acute PD. Some authors support that female gender is a risk factor for acute PD [27], while others do not [9,26,29]. Recurrent PD is a frequent sequel after primary PD, which affects physical activity and impairs the quality of life of the patient [8]. Fithian et al. reported a risk of 49% for recurrent PD after conservative treatment of the primary PD and an increased risk for contralateral PD as well [28]. A family history of PD has been reported in 28% of patients with recurrent PD [30].

PD is a sports-related injury

Atkin et al. found that 72% of dislocations occurred during sports activities, while two-thirds of these injuries occurred during level 1 cutting or pivoting sports [9]. Waterman et al. reported that over half (51.9%) of all PDs occurred during athletic activities [31]. Consequently, PD in the otherwise healthy adolescent and young adult is in most cases a traumatic event. This trauma is usually encountered during vigorous athletic activities, similar to activities that lead to anterior cruciate ligament (ACL) injury [9]. The level of sports participation before surgery is rarely reported; however, a recent systematic review and meta-analysis by Platt et al. showed that 83.1% of the patients with PD were amateur/recreational athletes and only 16.9% of patients had competitive/organized athletic activity [32].

PD relative to other athletic knee injuries: the patella fails last

Several studies on athletes participating in a competitive level of sports indicate that the PFJ is extremely durable to athletic trauma compared with other knee structures (ligaments, menisci). PD is rare compared with other “popular” athletic knee injuries, such as ACL rupture, although both injuries occur after a similar mechanism of injury [9]. Majewski et al. documented 7,769 knee injuries in 6,434 patients over a 10-year period [33]. They found that 44.82% of knee injuries involved internal anatomic structures of the knee: ACL lesion (20.3%), medial meniscus lesion (10.8%), lateral meniscus lesion (3.7%), medial collateral ligament (MCL) lesion (7.9%), lateral collateral ligament (LCL) lesion (1.1%), and posterior cruciate ligament (PCL) lesion (0.65%). Only 3.3% of patients had a knee dislocation, mostly PD [33].

Evaluation of the patient with PD

Workup of the patient with PD has three aspects: the acute trauma on the PFJ, the underlying predisposing factors of patellofemoral instability, and the overall neurologic and musculoskeletal status of the patient. Acute traumatic PD is almost always accompanied by injury of the medial patellar restraints and occasionally by osteochondral fractures, which are evaluated first [27]. The next step is to study the preexisting anatomical flaws of the PFJ, which render the patella unstable within the trochlea [8]. Finally, the overall neurologic and musculoskeletal status of the patient has to be appreciated to determine systemic factors, which affect lower limb biomechanics and promote PD or to detect poor neuromuscular cooperation between the muscles of the torso and the limbs. A diagnostic approach for the patient with acute PD is suggested in Table 1. Acute patellar dislocators usually present a combination of pathologic factors. Precise evaluation of these factors is mandatory to select treatment and estimate the patient’s prognosis.

**Table 1:** The diagnostic approach of the patient with acute PD PD: patellar dislocation, PFJ: patellofemoral joint, PFD: patellofemoral dysplasia, VMO: vastus medialis obliquus, TT-TG: tibial-tuberosity to trochlear groove distance, MPFL: medial patellofemoral ligament, MQTFL: medial quadriceps-tendon femoral ligament, MPTL: medial patellotibial ligament, MPML: medial patellomeniscal ligament, TD: trochlear dysplasia

Table 1: The diagnostic approach of the patient with acute PD		
The acute trauma of the PFJ after acute PD	Patellofemoral dysplasia (PFD)	General musculoskeletal status of the patient
1. Medial patellar restraints rupture/insufficiency	1. VMO weakness/atrophy	1. Hip abductor weakness
a. Upper medial restraints	2. Patella alta	2. Lower limb biomechanics
i. MPFL	3. Patellar tilt	a. Valgus knee
ii. MQTFL	4. increased Q-angle, increased TT-TG distance	b. Knee Recurvatum
b. Lower medial restraints	5. Lateral retinaculum tightness	3. Malrotation of the lower limb (femoral-tibial torsion)
i. MPTL	6. TD	4. Hindfoot valgus-forefoot pronation
ii. MPML		5. Ligament laxity
2. Osteochondral fractures		6. Core instability

The traumatic impact of acute PD on the PFJ

The Medial Patellar Restraints

The term medial patellar restraints describes the ligaments that stabilize the medial side of the PFJ [7,34]. PD implies a movement of the patella beyond the ultimate tensile stress of the medial patellar stabilizers. Consequently, injury (avulsion, rupture, plastic deformation) of the medial patellar restraints is the hallmark of all acute PDs [11,12]. The medial patellar restraints are classified into upper and lower medial patellar restraints [34]. When the knee is extended, the lower part of the patella is in loose contact with the upper part of the femoral trochlea [3]. At 30⁰ of knee flexion, the patella enters the trochlear groove. At this point, MPFL is the main medial ligamentous stabilizer of the patella [1,5,7,35,36]. Beyond 30⁰ of knee flexion, the trochlea and the lower patellar restraints (medial patellotibial ligament (MPTL), medial patellomeniscal ligament (MPML)) take over medial patellar stability. After the patella enters the trochlea, the contribution of MPFL as a medial patellar stabilizer is less important [5,37].

The Upper Medial Patellar Restraints: The MPFL and the Medial Quadriceps-Tendon Femoral Ligament (MQTFL)

The upper medial patellar restraints include the MPFL and MQTFL [34,36]. MQTFL and MPFL are not distinct but are parts of the same structure. The origin of MQTFL is at the distal anterior extent of the adductor tubercle, while the origin of MPFL is in the groove (saddle, flat area, sulcus) between the adductor tubercle and the medial epicondyle [34,36]. MQTFL and MPFL insert into the superolateral patella and the proximal half of the medial patellar border, respectively [34,36]. However, this separate description of one solid structure is not practical, and in the clinical setting, only the term MPFL is used to describe the upper medial ligamentous stabilizers.

The MPFL is the primary medial ligamentous stabilizer of the patella during the first degrees of knee flexion (0-30⁰), until the patella enters the trochlear groove [1,5,7,35]. It provides 50-60% of the total restraining force against lateral PD [5,7,35]. This medial restraining function of MPFL is further strengthened by vastus medialis obliquus (VMO) contraction [35].

**Figure 4:**
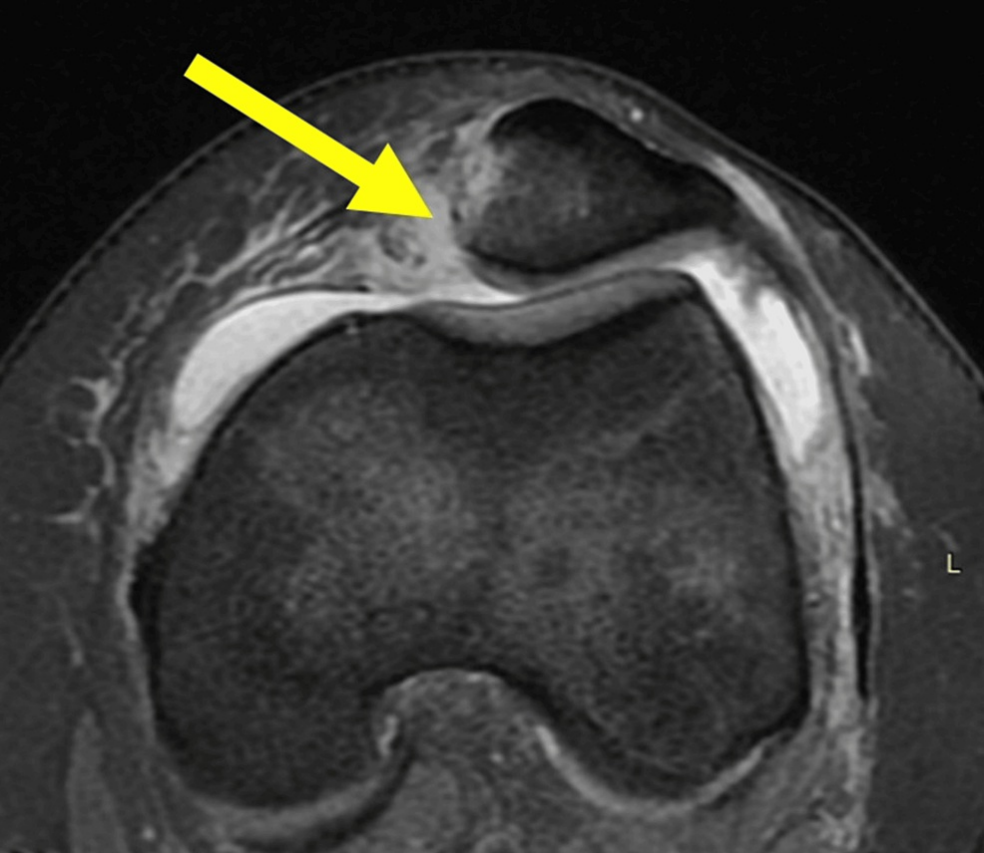
MRI of the left knee of a 13-year-old girl with acute PD Arrow indicates patellar avulsion of MPFL (arrow). Figure created by Panagiotis Samelis.

Almost all acute traumatic PDs, in either children or adults, are accompanied by complete MPFL injury (rupture, avulsion, plastic deformation, mixed) (Figure 4) [11,12,38]. In skeletally immature patients, the patellar attachment of MPFL is more frequently affected than the femoral attachment compared with adults [11,12].

**Figure 5:**
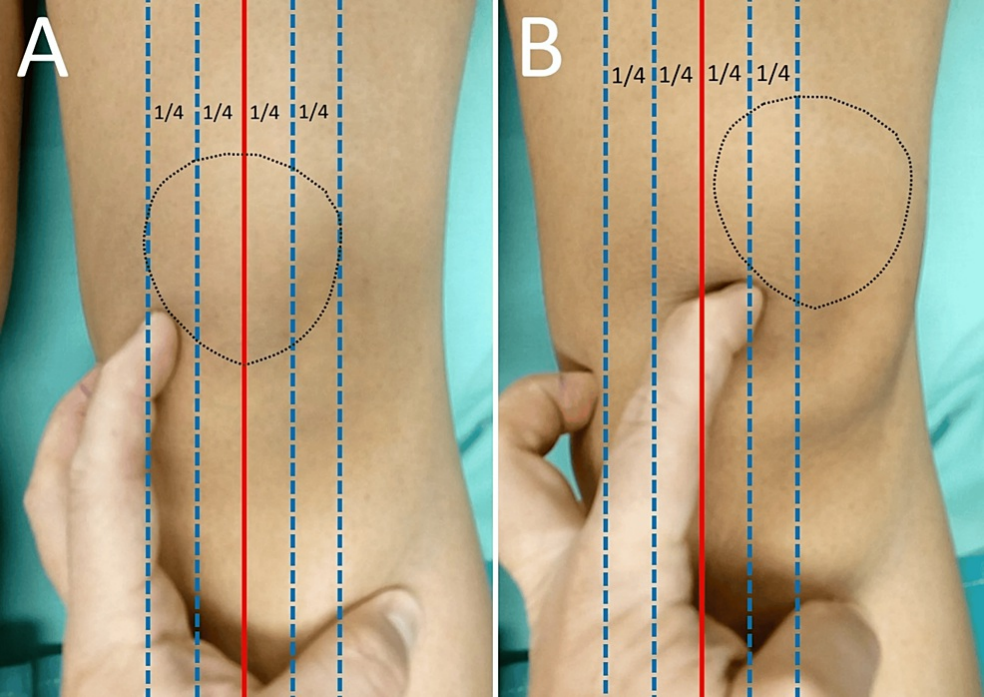
The lateral patellar glide test: apprehension and three quadrants of lateral glide of the right patella in a patient with a history of right PD (A) The knee is in 30⁰ of flexion and the quadriceps relaxed. The patella is divided into four longitudinal quartiles. (B) After being pushed laterally by the examiner, the patella glides laterally three quartiles of its width. In the patient with a history of PD and positive apprehension during the test, a positive lateral glide test indicates medial patellar restraints insufficiency. Figure created by Panagiotis Samelis.

The integrity of the medial restraints is assessed using the patellar glide test (Figure 5). This test is performed with the patient relaxed and the knee in 30⁰ of flexion. Lateral translation of the patella up to one quadrant of the patellar width or about one fingerbreadth is deemed normal and is usually not associated with patellar apprehension. More than three quadrants of lateral glide, if associated with apprehension, indicate lax medial patellar stabilizers [15].

The Lower Medial Patellar Restraints: MPTL and MPML

The lower medial patellar restraints include the MPML and MPTL [34-36]. MPTL and MPML are distinct ligamentous structures, histologically and biomechanically [36]. They share a common patellar attachment, which is located at the lower third of the medial border of the patella [34,36].

MPTL is a thin, broad ligament, which courses distally and inserts about 15 mm distal to the joint line and 15 mm medial to the patellar tendon [35]. On the anteroposterior X-ray view of the knee, the tibial attachment of MPTL corresponds to the lateral third of the medial tibial plateau or at the medial border of the base of the tibial spine [36]. In skeletally immature patients, the tibial attachment of MPTL is located above the proximal tibial physis [36]. MPML is a cordlike ligament [34], which courses more horizontally and posteriorly to MPTL and inserts medial to the anterior horn of the medial meniscus (or at the transition of the anterior horn and the body of the medial meniscus) [36]. MPML forms an angle of 15-30⁰ with the patellar tendon when the knee is extended [35]. MPTL contributes 24% and the MPML 13% of the restraining force against lateral PD [7,35]. This restraining force almost doubles at 90⁰ of knee flexion [5].

**Figure 6:**
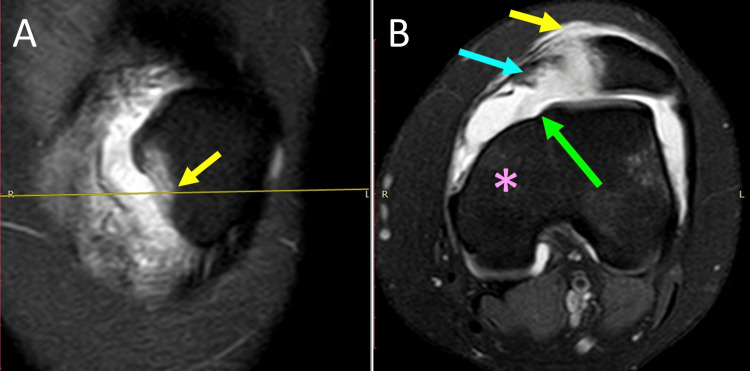
Isolated avulsion fracture of the inferomedial patellar retinaculum after acute left PD in a female adolescent athlete with Dejour-D TD and a hypoplastic medial femoral condyle MRI of the left knee: (A) Frontal plane: the arrow indicates the location of the avulsion fracture at the distal part of the medial patellar border. (B) Axial plane: patellar avulsion fracture (blue arrow), complete avulsion of distal medial restraints from the patella (yellow arrow), Dejour-D TD with a hypoplastic medial femoral condyle (asterisk), and a cliff-type appearance of the trochlea without a trochlear sulcus (green arrow). Figure published under Creative Commons Attribution License CC-BY 3.0 [40].

Studies have shown that in acute PD, the medial restraint injury was located at the inferomedial border of the patella, which corresponds to MPTL/MPML patellar attachment [37]. Thus, ligament injury in acute PD may start with MPML/MPTL rupture. This is explained biomechanically because the MPML/MPTL complex is in line with the quadriceps and is the first to oppose quadriceps contraction, which is the triggering force of PD [37,39]. Furthermore, in the case of concomitant severe TD, isolated MPTL/MPML rupture may lead to PD without simultaneous MPFL rupture (Figure 6) [40].

Osteochondral Fractures, Cartilage Injury, Bone Bruising, Avulsion Fractures, and Acute Knee Hemarthrosis

**Figure 7:**
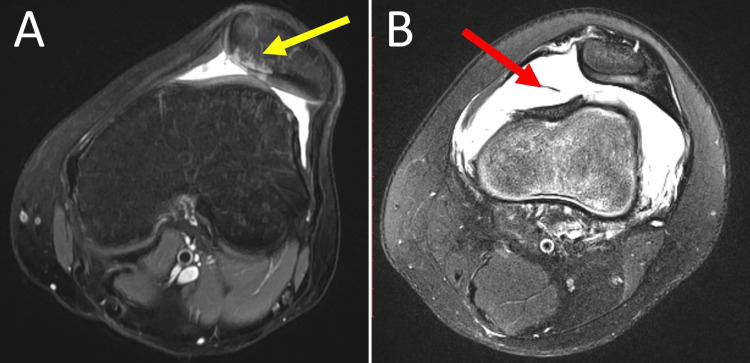
MRI of the knee in acute lateral PD (A) Arrow indicates bone bruising and chondral and osteochondral damage of the opposing articular surfaces. (B) Arrow indicates acute knee hematoma. Figure created by Panagiotis Samelis.

In acute PD, the patella jumps over the lateral trochlear wall and usually reduces spontaneously. At this point, the inferomedial aspect of the patella abuts against the anterolateral aspect of the lateral femoral condyle. This movement of the patella (dislocation followed by relocation) may cause bony and/or cartilage injury [27] between the opposing surfaces of the medial patella and the lateral trochlear wall [16]. The spectrum of this injury ranges from bone bruising to chondral and osteochondral injuries with free intraarticular fragments and acute knee hemarthrosis (Figure 7) [16,27].

Osteochondral fractures after acute PD are either avulsion fractures of the medial patellar restraints or free intraarticular chondral or osteochondral fragments [11,12,27]. A recent systematic review showed that chondral injury of the patella and the trochlea was evidenced on MRI in 85% and 47% of patients with acute PD, respectively, while free intra-articular fragments were observed in 11.5% of patients [38].

**Figure 8:**
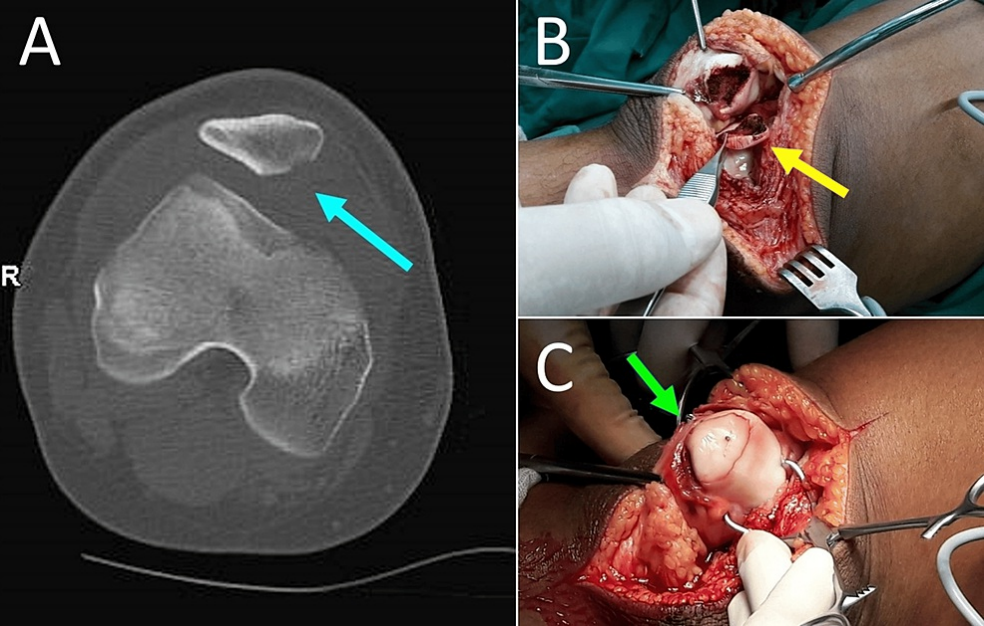
Treatment of osteochondral fracture after acute PD in an 11-year-old girl (A) Osteochondral fracture (blue arrow) of the medial facet and the central ridge of the patella following right PD. (B) The free osteochondral fragment was reduced (yellow arrow). (C) Fixation of the fragment with absorbable pins (green arrow). Figure created by Panagiotis Samelis.

Avulsion fractures of the patellar attachments of the medial patellar restraints may be observed in the context of ligamentous medial restraint injury [27,37]. Avulsion fractures should be fixed if they involve the medial patellar facet [41] or if the increased number and severity of patellar instability factors favor early surgery [40]. Free intra-articular osteochondral fragments should be reduced anatomically and fixed with absorbable pins; however, fragment fixation may be impossible for very small fragments (<1-1.5 cm), which are removed (Figure 8) [16].

Patellofemoral dysplasia: intrinsic PFJ pathology

Merchant et al. supported that PD never occurs on a normal PFJ. He coined the term “patellofemoral dysplasia” (PFD) to include all abnormally developed anatomic factors of the extensor mechanism of the knee, which predispose to patellar instability [8]. These factors are Vastus Medialis Obliquus atrophy, lateral retinaculum atrophy, patella alta, patellar tilt, knee valgus, the Q-angle, the tibial-tuberosity to trochlear groove distance, and TD [8].

VMO Weakness/Atrophy

VMO is the lower part of the vastus medialis (VM). VMO originates from the medial intermuscular septum of the femur and the tendon of the adductor magnus [34]. The fibers of VMO have a more horizontal orientation compared to the proximal part of the VM and are inserted into the middle layer of the trilaminar patellar insertion of the quadriceps and into the proximal half of the medial border of the patella [34].

**Figure 9:**
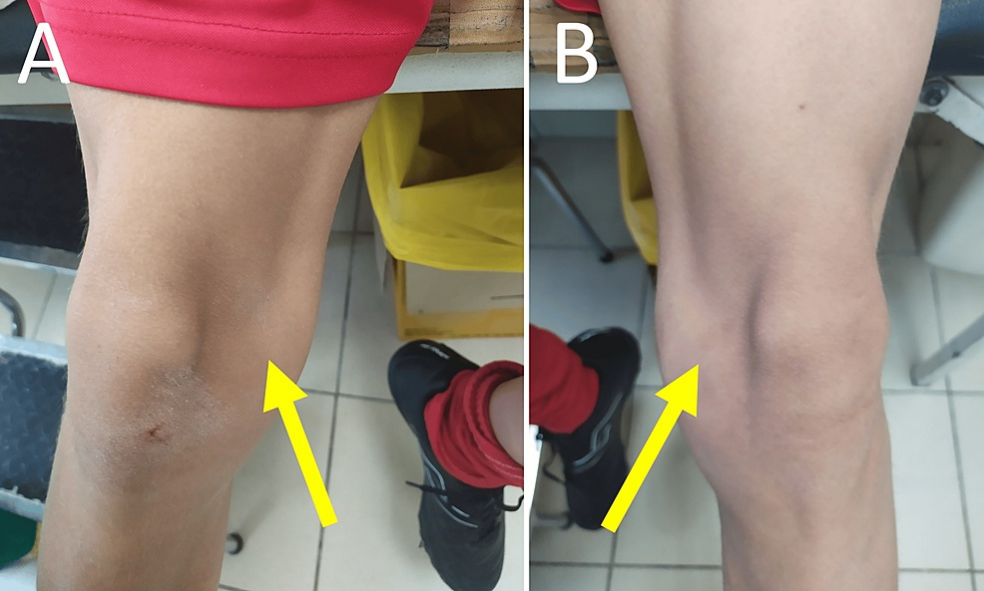
VMO atrophy in a 12-year-old boy with bilateral recurrent PD, knee valgus, ligament laxity, and positive family history of PD VMO of the right (A) and left (B) knee (arrows). Figure created by Panagiotis Samelis.

VMO deficiency in patients with PD is the result of a dysplastic VMO with a more proximal insertion on the patella [8]. VMO deficiency is clinically evident with resisted extension of the knee at 30⁰ (Figure 9). A deficient or relaxed VMO leads to a 30% reduction of lateral patellar stability at 20° of knee flexion [42]. In VMO-deficient knees, the bulk of the contracted VMO, which is normally seen at the medial edge of the patella, is absent [8]. It is not clear whether VMO deficiency is the result of dysplasia or atrophy secondary to pain [8].

Lateral Retinaculum Tightness

**Figure 10:**
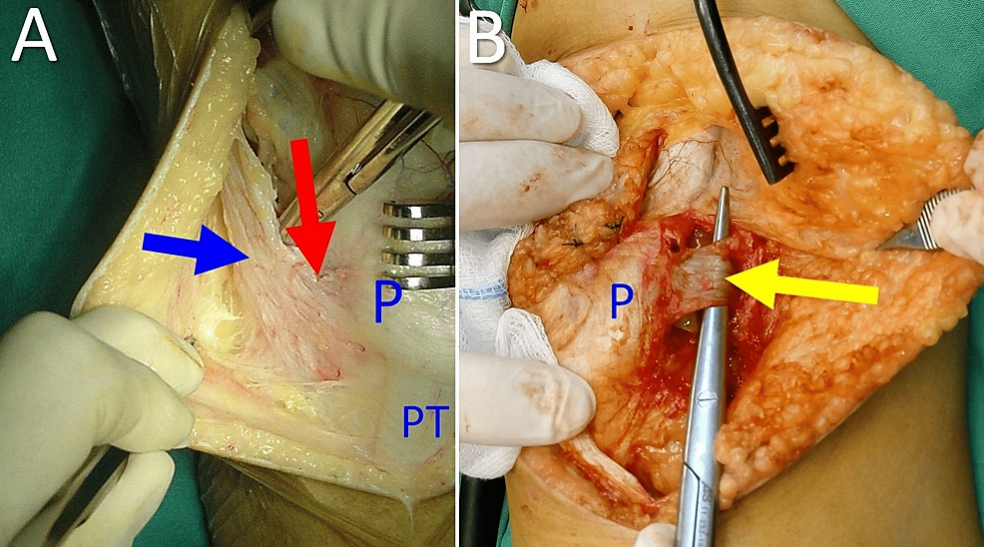
The lateral patellar retinacular structures (A) The IPB (red arrow) of the iliotibial tract (blue arrow). (B) The LPFL (yellow arrow). P: patella, PT: patellar tendon. Figure from Panagiotis Samelis.

The term lateral knee retinaculum describes all the fibrous structures, which stabilize the lateral aspect of the knee joint [7,43]. In relation to patellar stability, three distinct bands of connective tissue are associated with the lateral retinaculum of the knee. These bands form three ligaments: the iliopatellar band (IPB), the lateral patellofemoral ligament (LPFL), and the lateral patellotibial ligament (LPTL) [43]. The IPB lies on a more superficial level compared with the other two ligaments. The fibers of the IPB originate from the distal part of the iliotibial band and are inserted obliquely on the anterolateral aspect of the patella [43]. The LPFL and the LPTL are capsular thickenings [43]. The LPFL lies immediately deep into the IPB. The origin of the LPFL is distal and anterior to the lateral epicondyle, and its insertion is on the middle third of the lateral aspect of the patella [43]. The LPTL originates from the inferior aspect of the patellar insertion of the IPB and inserts anterior to Gerdy’s tubercle on the tibia and on the lateral meniscus (Figure 10).

Lateral retinaculum tightness is assessed with the patellar glide test. The knee is flexed 20-30⁰, the quadriceps is relaxed, and the patella is pushed medially. Medial displacement less than one-fourth of the patellar width is indicative of a tight lateral retinaculum, while a patellar glide ≥3 quartiles of the patellar width indicates a hypermobile patella [8,15].

Surgeons should bear in mind that lateral retinaculum tightness should be diagnosed with caution, since inconsiderate lateral retinacular release (LRR) may lead to iatrogenic medial patellar instability [43]. Therefore, LRR should be implemented only if the lateral retinaculum is definitely tight and only additional to other patellar stabilizing procedures.

Patella Alta

A high-riding patella, or patella alta, is a frequent finding in patients with PD. It is observed in 24-60% of acute PDs [9,44]. A higher position of the patella implies less contact with the trochlea, an abnormal position of the patella in the transverse plane (lateral shift), and an abnormal lateral tilt [45]. This leads to decreased stability of the PFJ during early knee flexion [45]. Therefore, patella alta is strongly associated with acute and recurrent patellar instability [8,44-46]. Patella alta is associated with higher stresses on the PFJ compared with normal individuals [47]. Consequently, patella alta is not only a risk factor for patellar instability but also a risk factor for patellofemoral pain and arthritis [47].

**Figure 11:**
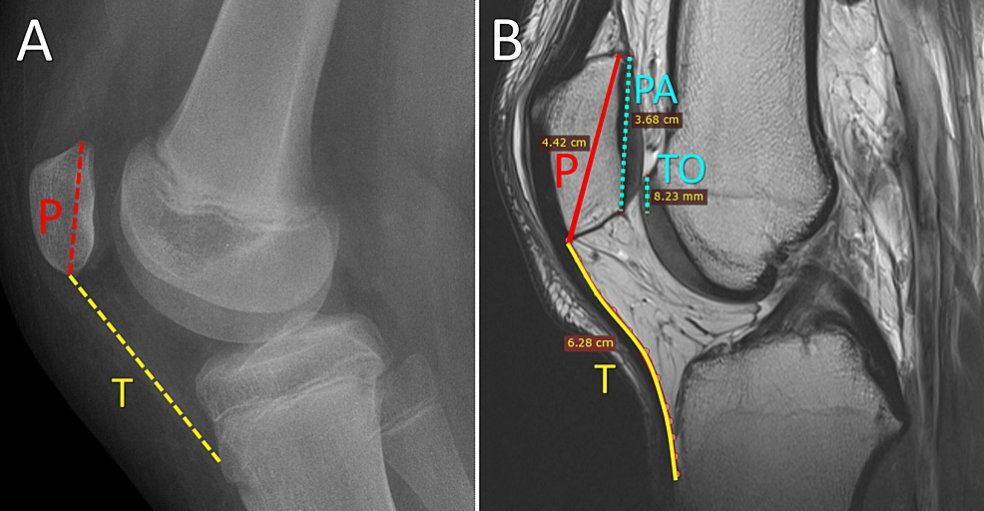
Assessment of patellar height (A) Patella alta of the right knee of an 11-year-old girl with PD. The Insall-Salvati ratio is the craniocaudal length of the patella (P) divided by the length of the patellar tendon (T) on the lateral plain X-ray view with the knee in extension. (B) The PTI in a 24-year-old elite football player with patellar tendinitis. The PTI is the ratio of the articulating part of the patella and the trochlea (TO) divided by the cephalocaudal length of the articular surface of the patella (PA) on the sagittal MRI of the extended right knee. Alternatively, the Insall-Salvati index may be measured on knee MRI as well, taking into account the slack of the patellar tendon (T) due to the extended knee. Figure created by Panagiotis Samelis.

Insall and Salvati described the homonymous index to measure the cephalocaudal position of the patella in the extensor mechanism of the knee [48]. They calculated the ratio of the length of the patellar tendon divided by the craniocaudal length of the patella in the lateral X-ray projection of the 30⁰ flexed knee. A ratio higher than 1.2 is indicative of patella alta [48]. Numerous other methods to measure patella alta have been proposed, suggesting that all have shortcomings. Biedert’s patellotrochlear index (PTI) measures the overlap between the articulating surfaces of the patella and the trochlea. It is measured on MRI with the knee in extension. PTI seems to be a more accurate representation of the patella-trochlea relationship. An index <12.5% is indicative of patella alta. Alternatively, the Insall-Salvati index may be measured on MRI; however, the slack of the patellar tendon has to be measured as well because the knee is in extension (Figure 11) [3].

**Figure 12:**
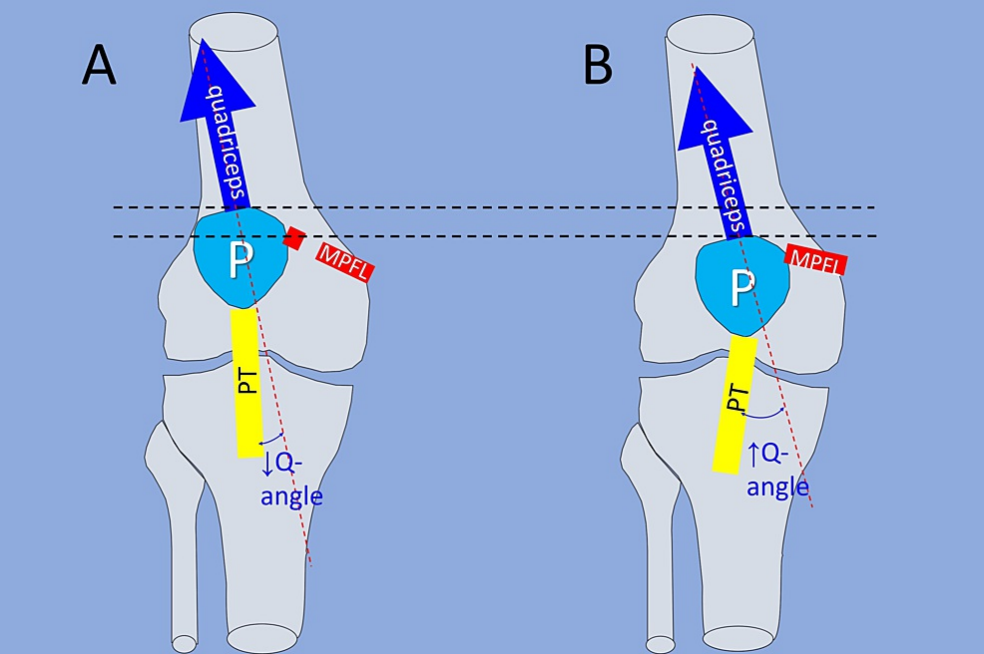
The relation of MPFL to the patellar height (A) MPFL rupture leads to patella alta and to a decrease of the original Q-angle due to lateral subluxation of the patella, and to relative straightening of the extensor mechanism of the knee. (B) MPFL reconstruction reverses this effect. MPFL: medial patellofemoral ligament (red), P: patella, PT: patellar tendon (yellow). Blue arrow: quadriceps contraction. Figure created by Panagiotis Samelis.

Interestingly, MPFL reconstruction is accompanied by a decrease in the patellar height [49]. This is secondary to a bow-string effect after MPFL rupture (Figure 12). Quadriceps contraction is unopposed by the MPFL, the patella subluxates laterally, and the quadriceps-patella-patellar tendon chain assumes a straighter orientation. Thus, MPFL rupture results in an even higher patella alta and a lower Q-angle compared to the pre-dislocation levels. MPFL reconstruction pulls the patella medially and distally, thereby decreasing the patellar height and increasing the Q-angle. Thus, in relation to patella alta, a surgical decision for tibial tubercle distalization is taken with caution, and certainly, anatomic MPFL reconstruction precedes any thought to restore the height of the patella.

Patellar Tilt

In the normal PFJ, the patella is centered into the trochlea, and both facets articulate with the respective trochlear walls. This relation does not change from 30⁰ to 90⁰ of flexion in normal knees [50]. In patients with patellofemoral instability, the patella quite often rests in a lateral position, manifested clinically or on the anteroposterior radiograph of the knee. This gives the impression that the congruity between the lateral patellar and the lateral trochlear facet is incomplete. The term patellar tilt is used to describe this abnormal position of the patella on the transverse plane of the PFJ [44,50].

Several methods have been used to measure the patellar tilt. The most popular measurements are the congruence angle of Merchant (measured on the tangential view of the Patella with the knees in 45⁰ of flexion) [50] and the patellar tilt angle (measured on CT scan of the PFJ with the knees in extension) [44].

The congruence angle is the angle formed between the bisector of the trochlea and a line that connects the central ridge of the patella and the deepest part of the trochlear sulcus [50]. In normal knees, the ridge of the patella is medial to the sulcus’ bisector, and the respective congruence angle is defined as negative [50]. If the patellar ridge is lateral to the sulcus’ bisector, the congruence angle is defined as positive. In normal knees, the congruence angle is always negative (points medially) and is on average -6⁰ (SD=11⁰) [50].

The patellar tilt according to Dejour et al. is measured on CT [44]. The axial cut of the knee at the midlevel of the patella is used [44]. The angle formed between the transverse axis of the patella and the posterior condylar line is the patellar tilt. In normal knees, the patellar tilt is <20⁰ [44]. Dejour et al. suggested that patellar tilt should be measured with and without quadriceps contraction to detect borderline cases with patellofemoral instability [13,44]. Increased patellar tilt is attributed to VMO deficiency and is correlated with TD [13,44]. Dejour et al. included patellar tilt among the major instability factors of the PFJ [13] and suggested that only a quadriceps advancement procedure may solve this problem [44].

The valgus Vector: The Q-Angle and the Tibial Tuberosity-Trochlear Groove Distance (TT-TG Distance)

Acute spontaneous PD is always lateral [51]. This is because normal knee alignment and normal knee movement create a laterally directed force vector that tends to dislocate the patella laterally [10]. This force is the result of the quadriceps contraction and the reaction force of the patellar ligament. The terms “the law of valgus” [10] or the “bowstring effect” of the extensor mechanism of the knee [52] were used to describe this phenomenon. The most popular measurements of the valgus vector force are the Q-angle and the TT-TG distance.

The (Static) Q-Angle

**Figure 13:**
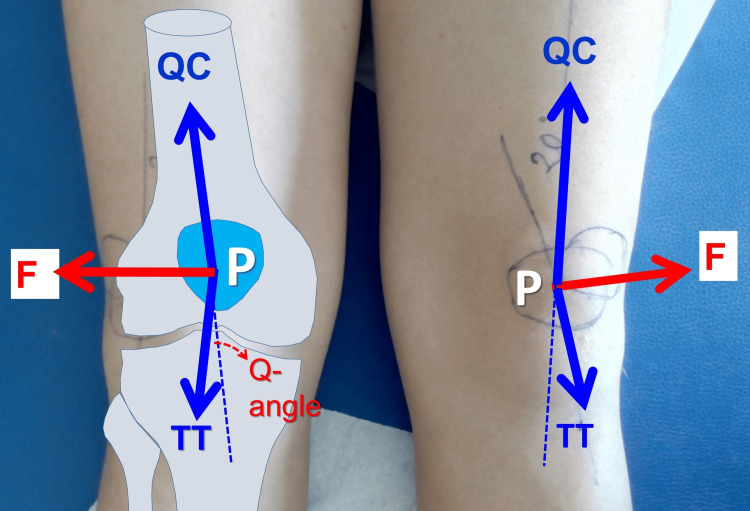
The impact of the quadriceps contraction on the stability of the patella The quadriceps contraction creates a laterally directed force (F, red arrow) that tends to dislocate the patella (P). A higher Q-angle implies a higher valgus vector and a higher laterally displacing force on the patella. P: patella, QC: quadriceps contraction, TT: tibial tubercle. Figure from Panagiotis Samelis.

The Q-angle is formed by the intersection of a line that connects the anterior superior iliac spine and the center of the patella and another line that spans the center of the patella and the tibial tuberosity (Figure 13) [52].

A higher Q-angle reflects increased laterally directed forces on the patella and higher contact pressures between the lateral patellar facet and the lateral trochlear wall [52]. Early studies reported that women have a higher Q-angle than men, due to their wider pelvis; however, later studies found similar Q-angles for men and women (13-15⁰) when adjusted for height [8,52]. The Q-angle should be measured on the fully extended knee after manual reduction of the patella into the trochlea to correct potential lateral subluxation of the patella (patellar tilt) and to measure the maximal Q-angle when the tibia is in the screw-home position [8].

The TT-TG Distance

Similar to the Q-angle, the TT-TG distance is also a measurement of the valgus vector force on the patella. The TT-TG distance is measured on CT or MRI of the extended knee by superimposing two transverse cuts, one at the deepest point of the trochlea and the other at the most prominent point of the tibial tuberosity [13,44,53]. The posterior condylar line is used as a reference. The TT-TG distance is the distance between the perpendicular to the posterior condylar line through the tibial tuberosity and the perpendicular through the trochlear groove. A TT-TG distance greater than 20 mm is a frequent finding in patients with patellofemoral instability [44]. Normal values of TT-TG distance are considered below 15 mm, borderline between 15 and 20 mm, and pathologic above 20mm [25,53]. In the clinical setting, a TT-TG distance greater than 20 mm is never an isolated finding in patients with patellar instability [25] and should not be considered an absolute indication for tibial tuberosity medialization [54].

TD

TD is used to describe the abnormal morphology of the femoral trochlea, which is a frequent finding in patients with PD. TD is the most important component of PFD and a major risk factor for acute and recurrent PD [44]. Furthermore, surgical treatment, such as MPFL reconstruction, may fail in cases with concomitant TD [22]. Main feature of TD is a shallow or flattened trochlea. A flattened trochlea fails to catch the patella during early knee flexion [25].

Several measurements and radiologic signs have been used to describe TD. The sulcus angle is among the oldest and most popular measurements to detect and quantify TD. The sulcus angle is measured on the tangential X-ray projection of the patella in about 30⁰ of knee flexion. It is formed between the highest points of the trochlear facets and the deepest point of the bony trochlear sulcus. This is the point where the patella enters the trochlear groove. In this projection, the sulcus angle should be less than 145⁰ [13,44,50]. According to Merchant et al., the normal sulcus angle is on average 138⁰ [50].

**Figure 14:**
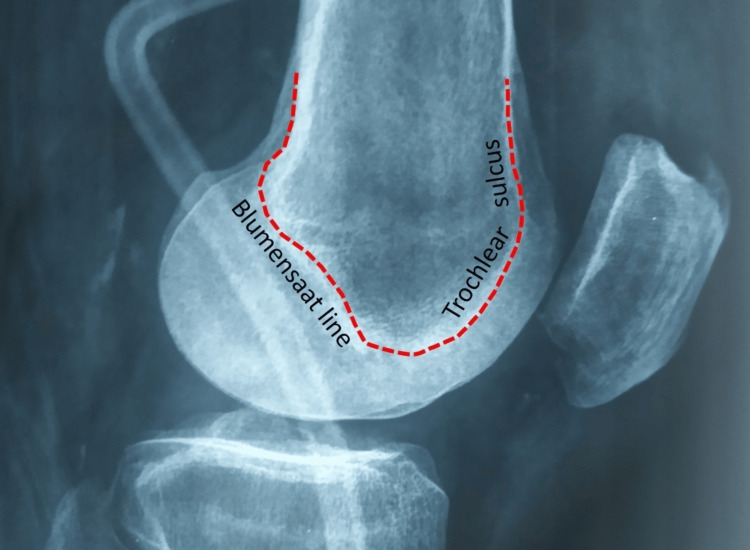
Assessment of TD on the true lateral plain X-ray of the knee The red line represents the intercondylar line of the distal femur. The anterior part of the intercondylar line is cartilaginous and represents the trochlear sulcus. The posterior continuation of the intercondylar line is osseous and represents the roof of the intercondylar notch. The posterior part of the intercondylar line is known as the Blumensaat line. Figure created by Panagiotis Samelis.

Henry and David Dejour et al. were the first to recognize various types of TD in patients with patellar instability [13,44]. They described three pathologic radiological signs of TD on the true lateral plain X-ray projection of the knee [13,44]: the crossing sign, the trochlear bump, and the double contour sign (Figure 14) [13,44].

The crossing sign indicates a shallow trochlea. It is observed in 96% of patients with a history of PD and only in 3% of healthy individuals [13,44]. On the true lateral X-ray projection of the knee, the sulcus of the trochlea (sulcus line) is represented by the anterior continuation of the Blumensaat line (the roof of the intercondylar notch) until it ends on the anterior surface of the femur. In normal knees, the sulcus line lies posteriorly relative to the anterior radiological contour of the condyles. This means that the trochlear sulcus is always below the level of the femoral condyles [44]. In TD, the sulcus line crosses the contour of one of both condyles, indicating that the trochlear sulcus is brought anteriorly relative to the femoral condyles. The crossing sign is diagnostic of a shallow or even a flat trochlea [44]. It is positive in 92.5% of the asymptomatic contralateral knees of patients with a history of unilateral PD, indicating that TD is a bilateral disease [44].

The trochlear bump (supratrochlear spur) is diagnostic of severe TD. In normal knees, the trochlear floor (sulcus line) ends smoothly on the anterior femoral cortex [13,44]. The term trochlear bump refers to a general prominence of the trochlea relative to the anterior cortex of the femur. When a trochlear bump is present, the proximal border of the trochlea ends abruptly on the anterior femoral cortex, creating a bony spur, termed the supratrochlear spur [13,44]. The trochlear bump is quantified on the true lateral X-ray of the knee by measuring the distance of the most anterior point of the trochlear groove from the continuation of the anterior femoral cortex. A trochlear bump greater than 3 mm is deemed pathologic for TD [44]. A higher trochlear bump indicates dysplasia of higher severity [44].

The double contour sign is created by a hypoplastic medial femoral condyle. On the true lateral knee X-ray, the contour of the hypoplastic medial condyle is projected quite posteriorly relative to the contour of the lateral condyle. On an axial CT or MRI cut, an abrupt continuation of the trochlea from the lateral to the medial femoral condyle is seen. This is referred to as the cliff pattern of the trochlea [13].

**Figure 15:**
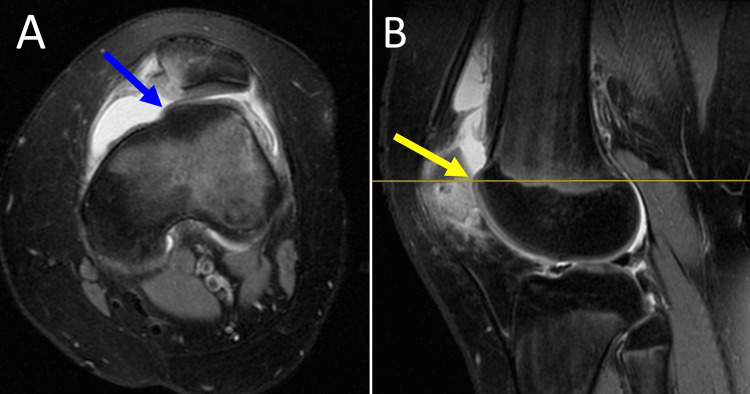
Type D TD of the left knee in a 14-year-old girl with first-time PD (A) Blue arrow indicates a cliff-type trochlea with a hypoplastic medial condyle. (B) Yellow arrow indicates a prominent trochlea, known as the trochlear bump. Figure created by Panagiotis Samelis.

According to the presence of not of these radiological signs and taking into account the axial morphology of the femoral condyles on CT or MRI, Dejour [13] classified TD into four types: A, B, C, and D (Table 2, Figure 15).

**Table 2:** Types of TD according to Dejour et al. [13,44]

	Type A	Type B	Type C	Type D
Crossing sign	+	+	+	+
Supracondylar spur		+		+
Double contour sign			+	+
CT/MRI of femoral condyles	Flat trochlea: Sulcus angle >145⁰	Flat trochlea: Sulcus angle >145⁰	Medial condyle hypoplasia	Medial condyle hypoplasia, cliff pattern

In a retrospective cohort study of 609 patients with first-time lateral PD, Sanders et al. found TD in 17.2% of patients [29]. Of these, 78% had type A, 18% had B, 2% had type C, and 2% had type D TD [29]. TD was a significant risk factor for recurrent PD during the follow-up period, followed by patella alta [29].

**Figure 16:**
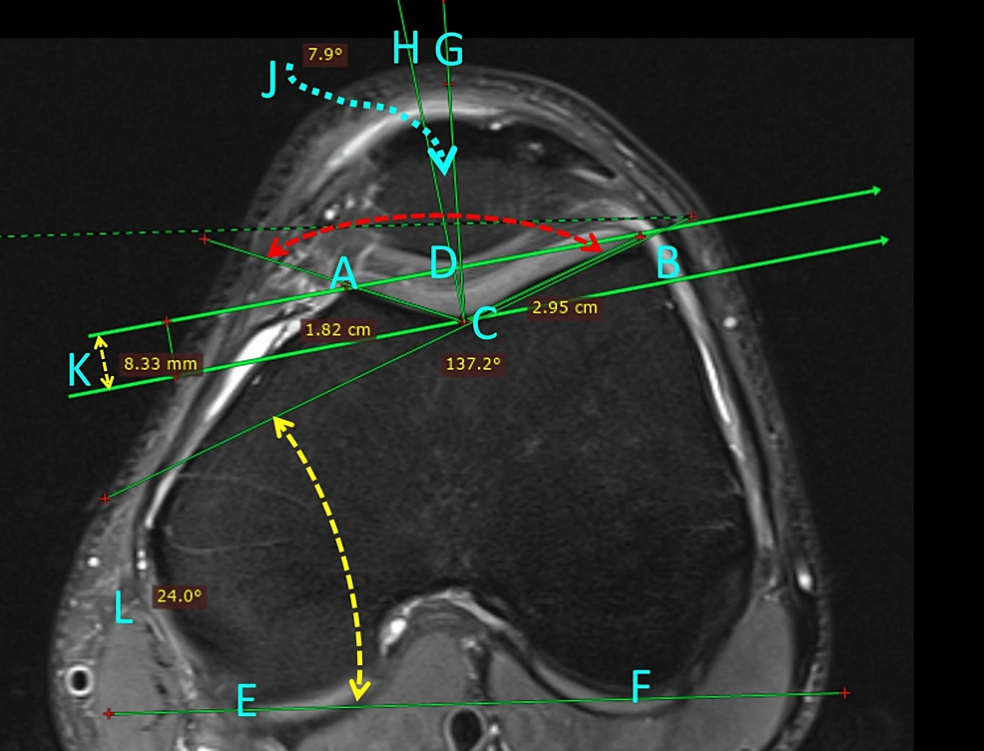
Measurements for TD Bony landmarks on axial MRI at the level of the fully shaped cartilaginous trochlea of the extended knee. (A) the highest point of the medial facet, (B) the highest point of the lateral facet, (C) the deepest point of the trochlear sulcus, (D) the apex of the median patellar ridge, (E) the posterior border of the medial femoral condyle, and (F) the posterior border of the lateral femoral condyle. The following measurements are made: AC: medial facet length, BC: lateral facet length, AC/BC: trochlear facet asymmetry, ACB: the sulcus angle (SA, red dotted line), CG: the bisector of the sulcus angle, DCG: the congruence angle (J, blue dotted line), the distance between the lines AB, and the line parallel through the point C is the sulcus/trochlear depth. The angle between the lines BC and EF is the lateral trochlear inclination (yellow dotted line). The RadiAnt-DICOM reader (Medixant, Poznań, Poland) was for the measurements. Figure created by Panagiotis Samelis.

Additional measurements based on CT or MRI have been described not only to diagnose but to quantify TD (Figure 16). However, to date, these measurements are not sufficient to point toward a specific surgical treatment. Therefore, Dejour’s methodology to detect and classify TD is more than enough to evaluate the patient with patellofemoral instability.

Torsional deformities of the lower limb

Femoral anteversion or antetorsion is the angle formed between the axis of the femoral neck and the line that connects the posterior aspect of the femoral condyles [44]. It may be more than 30⁰ in the newborn and gradually decreases to about 12⁰-15⁰ in the adult [55-58]. According to Dejour et al., femoral anteversion in the skeletally mature patient should be less than 20⁰ [44]. Femoral rotation (not torsion) is measured with the patient in the prone position and the knees flexed or with the patient supine and the hips flexed at 90⁰. Equal (about 40⁰) internal and external rotation of the femur with the hip flexed at 90⁰ indicates normal femoral antetorsion. Tibial torsion is the angle formed between the line that connects the posterior aspect of the tibial plateau and the bimalleolar axis [44]. External tibial torsion of about 25⁰ is deemed normal in asymptomatic adult individuals [56,59].

Torsional deformities of the femur and/or the tibia increase the dynamic knee valgus (DKV) and may predispose to patellofemoral pain and/or instability [56,59-61]. Excess femoral anteversion (>25-30°) increases DKV and promotes lateral patellar instability [13,44,61,62]. Even without PD, the increased lateral force vector on the patella leads to increased pressure at the lateral patellofemoral compartment and, in the long term, to patellofemoral osteoarthritis [22,24]. In the presence of an intact MPFL, 20⁰ of increased (above normal) internal femoral torsion is a significant risk factor for patellar instability [62], while in the presence of MPFL insufficiency (tear, laxity), only 10⁰ of increased femoral anteversion already represent a signiﬁcant risk factor [62]. Excess tibial external rotation is compensated by internal rotation of the hip [56,59]. Torsional deformities may compromise surgical treatment for patellar instability [62].

**Figure 17:**
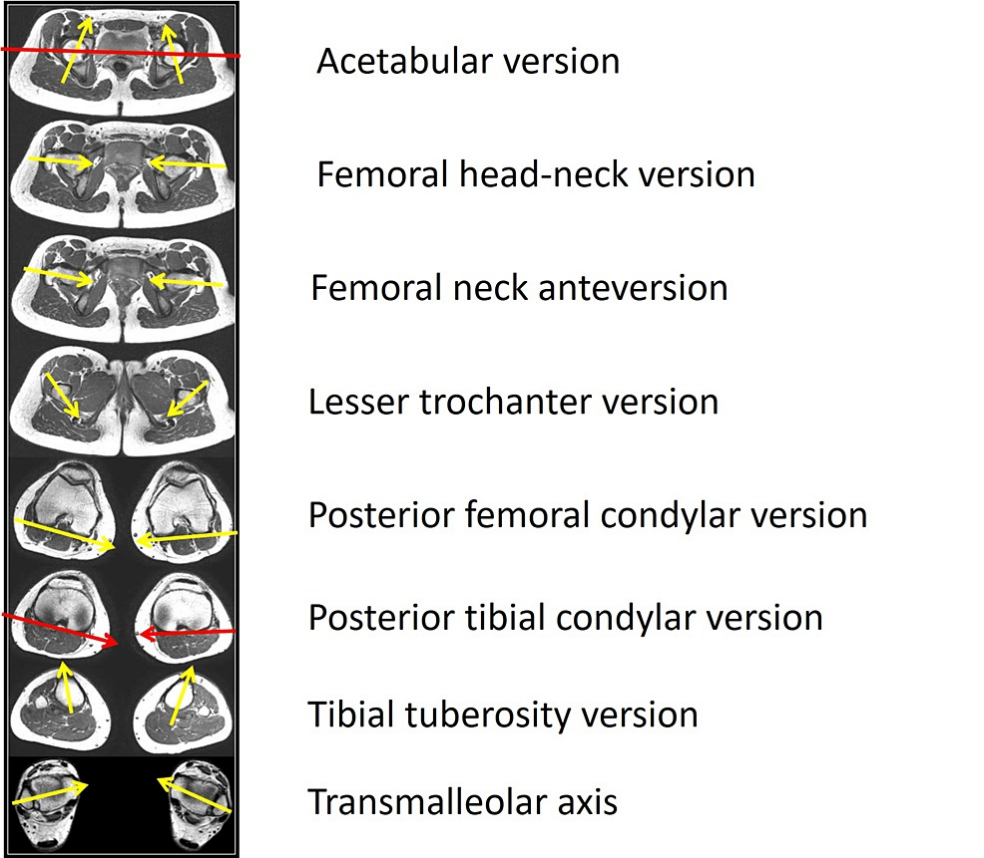
Long-limb MRI in an elite volleyball player with patellar tendinitis Sequential cuts at all levels of the lower limb are used. The torsional profile of the entire lower limb is assessed. Figure created by Panagiotis Samelis.

Evaluation of the torsional profile of the lower limbs is mandatory in patients with PFJ pain or instability. Torsional deformities of the lower limb are best studied on long-limb MRI or CT (Figure 17) [44,59,60].

The mechanical axis of the lower limb: the anatomical valgus

**Figure 18:**
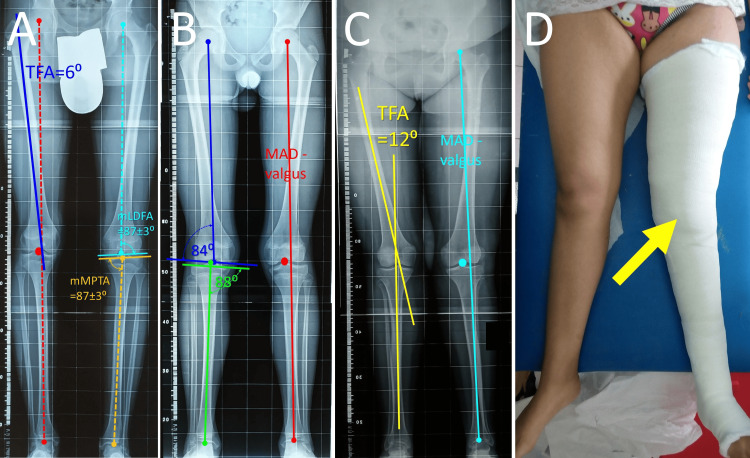
Mechanical axis assessment on ortho-roentgenogram of the lower limbs (A) Normal ortho-roentgenogram. The red dotted line is the mechanical axis of the lower limb. The dark blue line is the anatomical axis of the right femur. The mLDFA (mechanical lateral distal femoral angle) is the angle formed between the mechanical axis of the femur (dotted light-blue line) and the tangential to the femoral condyles line (continuous light-blue line). The mMPTA (mechanical medial proximal tibial angle) is the angle formed between the mechanical axis of the tibia (orange dotted line) and the tangential to the tibial condyles (continuous orange line). (B) A patient with recurrent PD. The mechanical axis of the lower limb (red line) is lateral to the center of the knee (red dot) indicating a valgus mechanical axis deviation (valgus-MAD) of the knees. Measurement of the mLDFA and the mMPTA indicate that the distal femur is the predominant cause of MAD. (C) A patient with acute lateral PD. The tibiofemoral angle (TFA) is the angle between the anatomical axis of the femur and the tibia (yellow lines). It is also useful to quantify knee valgus. A valgus-MAD is evident as well (light-blue line and dot). (D) The patient of Figure C. Marked knee-valgus is obvious on clinical examination. The patient received acute repair of a patellar avulsion of MPFL, and the knee was immobilized in a long-leg cast (yellow arrow). A, B, C: Figures created by Panagiotis Samelis. D: Figure published under Creative Commons Attribution License CC-BY 3.0 [40].

The axial alignment of the lower limb is best assessed on the ortho-roentgenogram (AP long-limb standing X-ray projection). It is obtained with the knees and the medial malleoli in slight apposition (no contact), while the patellae are facing forward. The mechanical axis of the lower limb (axis of Mikulicz), that is, the line that spans the center of the femoral head and the middle of the talar dome, is drawn [58,60]. Normally, the mechanical axis runs between the tibial spines or through the medial tibial spine, about 5-10 mm from the center of the knee joint or within 4±2 mm medial to the knee center [63]. Mechanical axis deviation (MAD) >15 mm medial to the knee center is associated with clinically significant genu varum, while a deviation >10 mm lateral to the knee center is associated with clinically significant genu valgum [60,63]. Other authors classify MAD into three zones on either side of the tibial spine. Zone 0 is in the intercondylar notch, zone 1 in the inner half of the articular surface of the tibial condyle (medial or lateral), zone 2 in the outer half of the tibial condyle, and zone 3 outside of the articulating surface of the tibial condyle [55,60]. If the mechanical axis goes through zone 1, the MAD is considered minor and is probably within the normal range. If the mechanical axis runs through zone 2, the MAD is relatively worse and has to be followed for potential surgical treatment. A MAD within zone 3 is considered severe and is an indication for surgical treatment [58]. Further assessment of a pathologic mechanical axis deviation includes measurement of the tibiofemoral angle (TFA), the mechanical lateral distal femoral angle (mLDFA), and the mechanical medial proximal tibial angle (mMPTA). The TFA is the angle formed between the anatomical axis of the femur and the axis of the tibia on the frontal plane. It normally is <7⁰ with lateral deviation of the tibia relative to the femur [63]. The mLDFA is the angle formed laterally between the mechanical axis of the femur (center of the femoral head to the center of the femoral condyles) and the tangent to the femoral condyles. The mMPTA is the angle formed medially between the axis of the tibia and the tangent to the tibial plateau. Both angles are normally 87⁰±3⁰ (Figure 18) [55,63].

The DKV: hip muscle atrophy

The originally described Q-angle is a static measurement (static Q-angle) on the frontal plane between bony landmarks of the unloaded lower limb [64]. However, closed kinetic chain activities, such as landing, pivoting, or deceleration maneuvers during sports, lead to a medial displacement of the knee [65,66]. This is the result of hip adduction and internal rotation of the femur under the patella with subsequent medial movement of the knee, relative to the foot, which is planted on the ground (closed kinetic chain) [65]. Medial movement of the knee leads to abduction of the tibia and foot pronation [65]. This phenomenon is the DKV [65]. Increased DKV implies increased strain on the ligaments, which resist knee valgus, such as the MCL, the ACL, and the MPFL [65]. Consequently, the (static) Q-angle underestimates the true lateral force vector created by quadriceps contraction, indicating that the actual quadriceps contraction angle is higher than the static Q-angle [66]. Strengthening of hip extensors, abductors, and external rotators decreases DKV and is an important part of physical therapy in patients with patellofemoral pain or instability [65,66].

Generalized ligament laxity-isolated patellar hypermobility

**Figure 19:**
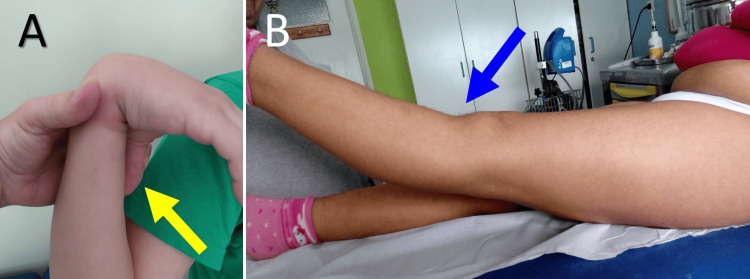
Clinical signs of joint hyperlaxity Positive thumb to forearm test (A) and knee recurvatum (B) in two patients with recurrent PD and joint hyperlaxity. Figure created by Panagiotis Samelis.

Generalized ligament laxity and native patellar hypermobility are factors associated with increased risk for patellofemoral instability [15]. The Beighton criteria are used to assess generalized ligament laxity [15]. Thus, elbow hyperextension >10⁰, thumb-to-forearm apposition, knee recurvatum >10⁰, little finger extension >90⁰, and forward trunk bending with extended knees until the palms touch the ground are signs indicating generalized ligament laxity. On the other hand, three or more quartiles of lateral patellar glide indicate isolated patellar hypermobility. Both situations may predispose to PFJ instability (Figure 19) [15].

Foot pronation

Foot pronation (pes planus, rearfoot valgus, foot eversion, subtalar joint pronation) is defined as the medial inclination of the foot around the axis of the subtalar joint [67]. Subtalar joint pronation occurs normally during the first 30% of the gait cycle and is accompanied by 6-10⁰ of tibial internal rotation because the tibia follows the internal rotation of the talar dome [68]. Thus, tibial rotation coordinates subtalar (pronation/supination) and knee joint motion (flexion/extension) in order to control the ground reaction forces during walking [69]. Foot hyperpronation may disrupt the normal rotation of the tibia and impair PFJ biomechanics, causing symptoms from pain to instability [69]. In relation to the pelvis, foot pronation may increase hip internal rotation. Hip internal rotation decreases the lever arm of the abductors, leading to abductor muscle fatigue and increased hip adduction, which, in the presence of weak hip abductors, increases the DKV [68].

Core stability

Core stability refers to the coordinated function of the muscles of the torso (abdominal muscles, muscles around the spinal column, the diaphragm, muscles of the pelvis), which stabilize the spine in order to transmit force to the limbs during athletic activity. Core stability is essential to maximize efficient athletic function. Poor core stability may predispose to athletic trauma of the lower limb, including the PFJ. Therefore, hip and core strengthening is a key element in patients with patellofemoral pain and instability [65,66,70].

Treatment of PD

Treatment of PD aims to provide a stable, painless, and functional PFJ and to prevent recurrent dislocation in the long term [71-74]. Traditionally, the first (acute, primary) incidence of PD is treated conservatively. The goal of treatment is to allow healing of the medial patellar stabilizers, to restore patellar stability and to strengthen the dynamic stabilizers of the patella and the hip muscles to control DKV [9]. A staged rehabilitation protocol is recommended to optimize recovery and return to pre-injury levels of activity. The patella is reduced and the knee is splinted in extension for three weeks. After the third week, early active motion and VMO and quadriceps strengthening are advised. The patient is encouraged to gradually return to his previous level of activity [8].

Surgical intervention on the first incident of PD is recommended only in the case of osteochondral fragments, which are reduced and fixed or removed if too small for secure fixation [9,16]. Surgical treatment of the underlying predisposing factors of patellar instability is usually reserved for recurrent PD. A significant number of surgical procedures have been described. The selection of one technique over the other or the combination of surgical procedures depends on the detection and evaluation of all factors of patellar instability of the individual patient. The presence of open physes alters the treatment plan by ruling out osseous procedures [71]. On the other hand, restoration of the patella-trochlea contact in the skeletally immature patient seems to be necessary for the normal development of the PFJ [72-74].

Decision-making for surgical treatment of PD is not straightforward. For example, patients with an ACL tear almost always receive some type of ACL reconstruction on an anatomically more or less normal knee. On the other hand, PD presents not a single pathology, and in most cases, PD refers to skeletally immature patients [9,26-28]. The selection of surgical treatment for PD is much more complex, and the correction of the multiple aspects of PFD is almost impossible. Since MPFL injury is almost always present after PD [11,12], anatomic restoration of MPFL is the primary surgical treatment of PD and should be preferred over non-anatomic extensor-mechanism realignment procedures [18]. Further surgical intervention to correct factors of PFD depends on the severity and the impact of each factor on the risk of recurrent PD [44]. Osseous procedures are allowed only after skeletal maturity. In the skeletally mature patient, tibial tuberosity medialization is indicated with a TT-TG distance >20 mm, while tibial tuberosity distalization is considered in the case of patella alta (Insall-Salvati >1.5 [22]). Deepening sulcus trochleoplasty is attempted with caution, in severe cases of TD, especially the Dejour B and D type, while the Albee procedure (lateral facet elevation) may be attempted in the Dejour type C TD [75].

**Figure 20:**
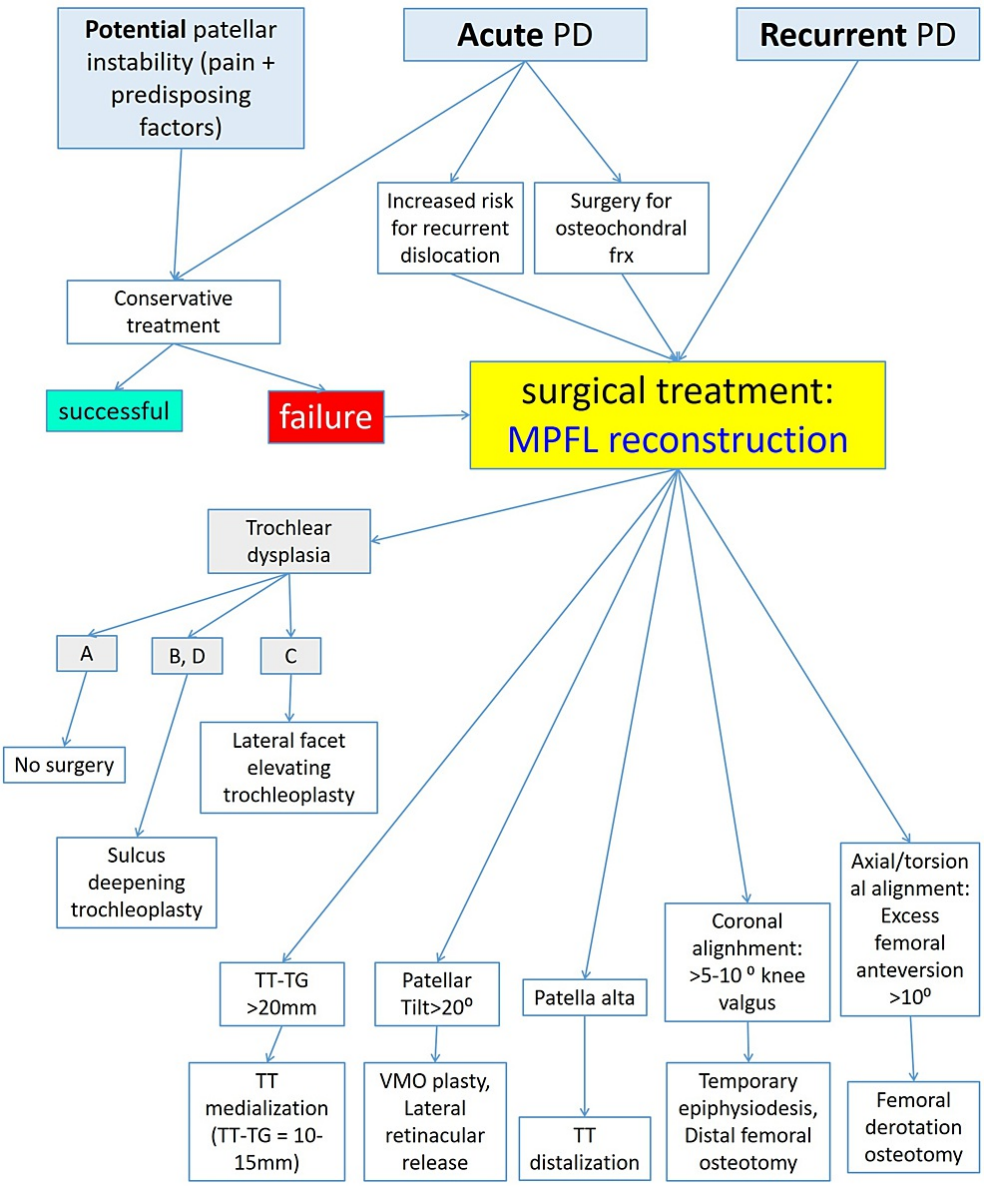
Decision-making algorithm for the treatment of PD PD: patellar dislocation, MPFL: medial patellofemoral ligament, TT: tibial tuberosity, TG: trochlear groove. Figure created by Panagiotis Samelis.

Surgical treatment is individualized according to the special characteristics of each patient with patellar instability (Figure 20).

Non-anatomic vs. anatomic surgical techniques for PD

Surgical procedures to treat PD are classified into non-anatomic vs. anatomic procedures.

**Figure 21:**
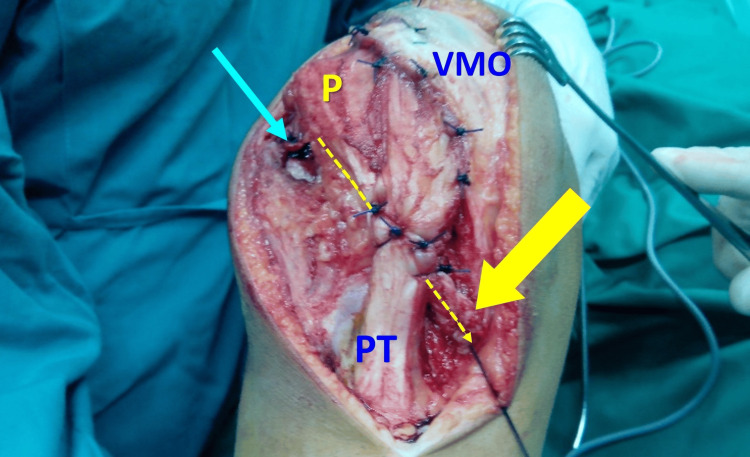
The Roux-Goldthwait procedure, VMO advancement, and lateral release (blue arrow) in an 11-year-old boy with recurrent PD, knee valgus, and lateral retinaculum tightness The lateral half of the patellar tendon (dotted arrow) is transferred medially under the medial half and secured on the medial aspect of the tibia (arrow). P: patella, VMO: vastus medialis obliquus, PT: patellar tendon. Figure created by Panagiotis Samelis.

Non-anatomic procedures (extensor mechanism realignment procedures) do not restore the local abnormal anatomy of the PFJ of the individual patient but rather attempt to centralize the patella inside the trochlear groove. Patellar stability is expected to follow after the restoration of the PFJ congruity. Non-anatomic procedures have been widely used in the past [14]. Such procedures are the Roux-Goldthwait procedure (distal patellar realignment by means of medial transfer of the lateral half of the patellar tendon) (Figure 21), lateral retinacular release, the Insall procedure (proximal patellar realignment with medial reefing and VMO advancement), various tibial tubercle osteotomies (anteriorization, medialization, anteromedialization), and several other procedures and modifications, which are performed isolated or in combination [20].

In the past, non-anatomic procedures were used indiscriminately on all patients with recurrent patellar instability. Dejour et al. rationalized the workup and treatment of patellar instability because they focused on the distinct patellar instability factors [13,44]. Treatment of patellar instability is individualized for each patient, based on the number and severity of the abnormal anatomic factors of her/his particular PFJ [13,44]. Anatomic procedures gained popularity among surgeons over the years [14]. MPFL reconstruction is the gold-standard treatment for PD, leading to lower rates of redislocation compared with non-anatomic techniques. Varisation and torsional osteotomies are increasingly used to augment MPFL reconstruction as well [14].

Surgical considerations for MPFL

Graft isometry during knee motion is a prerequisite for successful MPFL reconstruction [76]. A non-isometric MPFL graft leads to restriction of patellar motion, increased pressures on the medial PFJ, failure of MPFL reconstruction, and recurrent dislocation [76].

Recent anatomic studies locate the femoral origin of MPFL at the groove between the adductor tubercle and the medial epicondyle [34,76,77]. The femoral attachment of MPFL is determined intraoperatively, either radiographically [78] or after surgical exploration of the femoral footprint of MPFL on the medial surface of the medial femoral condyle [34]. According to Schoettle et al., three lines are drawn on a true-lateral knee radiograph (perfect overlap of the posterior contours of the femoral condyles): the posterior cortex line of the femur and two lines perpendicular to the posterior cortex line. One of these perpendiculars intersects the upper limit of the Blumensaat line and the other the posterior upper end of the femoral condyles [78]. The femoral origin of MPFL is located between the two perpendicular lines, just anterior to the posterior cortex line of the femur [78]. The accuracy of the radiographic location of the femoral attachment of MPFL is questioned. True lateral knee radiographs are difficult to obtain intraoperatively [34,76]. Moreover, radiographic landmarks do not reflect anatomic variability between patients [34,76]. Therefore, several authors prefer surgical exploration to define the femoral attachment of MPFL using the tendon of the adductor magnus as a landmark to locate the adductor tubercle [34,76,77].

The patellar attachment of the MPFL is fan-shaped. It is located at the upper two-thirds or the upper half of the medial border of the patella [7,34,35]. Just proximal to the patellar attachment, muscle fibers of VMO insert directly into the MPFL. It has been suggested that VMO contraction tethers MPFL and increases the posteromedial force that holds the patella into the trochlear groove [35].

In skeletally immature patients, the adductor tubercle and the medial epicondyle, and hence, the femoral attachment of MPFL are distal to the distal femoral physis [71]. When MPFL reconstruction is attempted in skeletally immature patients, if the graft is attached proximal to the physis, ongoing growth will lead to proximal migration of the femoral attachment of MPFL relative to the femoral condyles. This will probably lead to asymmetric forces on the PFJ and to the failure of the reconstruction [79]. Furthermore, the distal femoral physis is not even but wavy. Thus the tunnel for the femoral attachment of MPFL is drilled into the medial femoral condyle in an oblique caudal direction to spare the distal femoral physis [71].

**Figure 22:**
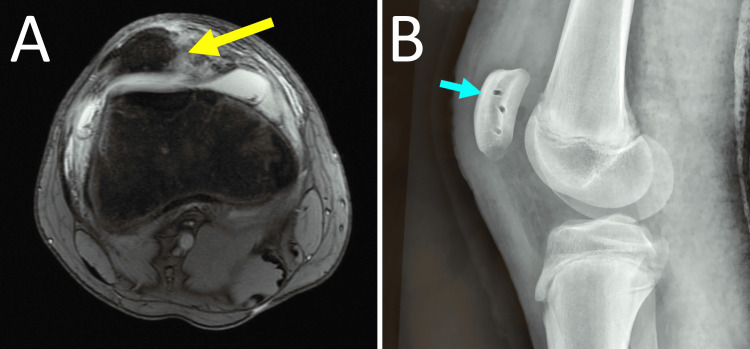
MPFL patellar avulsion and repair in an adolescent with acute PD (A) Patellar avulsion of MPFL (yellow arrow). (B) Transosseous sutures through the patella (blue arrow). Figure created by Panagiotis Samelis.

In the case of surgery for acute primary PD all studies agree, that MPFL reconstruction is superior to MPFL repair in reducing the rate of redislocation [80]. This is because MPFL injury is in many cases multifocal, or the ligament sustains permanent plastic deformation after the first dislocation [11,12,81]. Thus, the injured MPFL is practically not suitable for repair but rather for reconstruction [81]. However, acute MPFL repair may be selected in the case of patellar avulsion of MPFL, which involves the articular surface of the patella (Figure 22).

Surgical considerations for MPTL/MPML

MPTL reconstruction cannot substitute MPFL reconstruction; however, it is useful as a secondary procedure to provide additional stability [36,37,39]. Combined MPTL and MPFL reconstruction has better outcomes than isolated MPFL reconstruction without increasing the complication rates [36]. MPTL reconstruction may decrease the need for osseous procedures [39]. This is particularly important in skeletally immature patients in which osseous procedures could harm the growth plates around the knee [36]. A positive Garth test (with the knee in terminal extension, superolateral subluxation of the patella occurs when manual pressure is applied on the inferomedial patella in a superolateral direction) is also an indication for simultaneous reconstruction of the lower medial patellar restraints along with MPFL reconstruction [36,39]. In case of combined MPFL or MPTL reconstruction in skeletally immature patients, care should be taken to secure the graft on the distal femoral metaphysis (MPFL) [71] or the proximal tibial metaphysis (MPTL) [36], to avoid growth disturbance because of tethering or injury of the physis.

Surgical considerations in the skeletally immature patient

Skeletal maturity and remaining growth are important factors in decision-making for surgery for patients with PF instability [75]. Osseous procedures, such as tibial tuberosity transfer or trochlear groove deepening, are not used in patients with open growth plates to avoid premature distal femur or proximal tibia epiphysiodesis and subsequent lower limb deformity [71]. Practically, the majority of PD patients, which are skeletally immature children and adolescents, receive only soft tissue surgery, such as the Roux-Goldthwait procedure, while MPFL reconstruction, which is the most important medial patellar restraint, is performed with caution to spare the distal femoral physis during the preparation of the femoral attachment of the MPFL [71,75]. Non-osseous anatomical MPFL reconstruction (non-osseous modifications) or non-anatomical Roux-Goldthwait procedure are indicated in isolated MPFL lesions in children. In skeletally immature patients with marked patella alta (Insall-Salvati >1.5), the imbrication of the patellar tendon (shortening by folding and resuturing the patellar tendon) may be used as an adjunct procedure [75,83].

In skeletally immature patients, isolated MPFL reconstruction yields generally good results, with redislocation rates between 0-9.3% [71,79]. It has been suggested that surgical stabilization of the patella may improve trochlear morphology in children with underlying TD [72]. Increasing evidence supports isolated MPFL reconstruction without additional intervention on preexisting bony abnormalities, such as patella alta or TD [79,84,85].

Surgical considerations for torsional and mechanical axis deformities in patients with patellar instability

Torsional and mechanical axis abnormalities of the lower limbs are frequent findings in patients with PFJ instability [60]; however, they are not specific for patellar instability. Furthermore, there is a wide overlap in the torsional and/or axial profile of the lower limbs between patients with patellofemoral instability and healthy controls [61]. Moreover, TD may coexist with torsional or axial deformities and increased TT-TG distance [13,44]. Therefore, torsional/axial deformities of the lower limbs are not an absolute indication for corrective surgery in patients with PFJ instability [44]. Before any decision for surgical intervention, the entire extensor mechanism of the knee should be assessed to determine if the torsional/axial deformity is among the dominant factors of patellar instability to be addressed [86].

Femoral derotation osteotomies (FDO) should be reserved only for symptomatic patients and not for cosmetic reasons (e.g., in-toeing, inward pointing patellae) [86,87]. FDO should be used as an additional procedure in combination with MPFL reconstruction or other bony procedures in patients with symptomatic patellofemoral instability and femoral anteversion >20-30⁰ [13,22,44,62,88,89] or if femoral external rotation is less than 15⁰ [61]. The aim of the osteotomy is to restore the femoral anteversion to normal (15⁰) in order to correct the transverse knee alignment [61,87] and to avoid tensioning the medial patellar stabilizers [62].

Normal tibial (external) torsion is about 24-30⁰ [59]. It has been suggested that tibial derotation (supramalleolar) osteotomy should be performed if the thigh-foot angle is greater than 30⁰ on clinical examination [59]. Decisions on the amount of correction may be more difficult if CT measurements of the excess tibial torsion are used. Thresholds of 5-20⁰ above normal external tibial torsion in symptomatic patients have been proposed [59]. Server et al. performed proximal tibial osteotomy in symptomatic patients with lateral tibial torsion >30⁰ and a normal to moderate femoral antetorsion (<25⁰) [56].

Albersheim et al. suggested a practical guideline to decide on femoral and/or tibial osteotomy to correct excessive femoral or tibial rotation in patients with PFJ symptoms [90]. The authors use a surgical algorithm based on groupings of 10⁰ of axial plane abnormality. Zero to 20⁰ of torsional malalignment is considered normal, and no derotation osteotomy is needed. Twenty to 30⁰ of torsions are deemed abnormal but rarely warrant surgical treatment and only if the rotational deformity contributes significantly to PF pathology. Thirty to 40⁰ are more likely surgical candidates, depending on the PF pathology. More than 40⁰ in patients with PF symptoms almost always receive derotational surgery [90]. This algorithm applies to both femoral and tibial rotation. Concomitant torsional deformity of both bones is frequent and may have to be treated simultaneously [90]. Furthermore, a femoral derotational osteotomy will lead to tibial external rotation, or exacerbate preexisting tibial external rotation [90]. Therefore, surgical treatment of torsional deformities is decided with caution and after a detailed interpretation of the underlying patellofemoral pathology [60].

Lateral PD and knee valgus are inseparable [51]. Therefore, knee valgus correction should be seriously considered as an early surgical option in patients with PFJ instability and valgus MAD. In skeletally mature patients, a varization osteotomy is the only option to correct knee valgus; however, in skeletally immature patients, guided growth techniques are usually applied [51,61,88]. Especially in younger children (<10 years old, before the growth spurt) with concomitant TD, a MAD correction could improve PFJ biomechanics and potentially promote some trochlear remodeling during the remaining growth [4,51,73,91]. Temporary medial knee epiphysiodesis (distal femur and/or proximal tibia) using eight plates (tension-band taping) is a safe, minimally invasive technique with low perioperative morbidity [51], which can be used for the correction of knee valgus >5-10⁰ in children with at least 12 months of remaining growth [51,91].

Surgical vs. conservative treatment of acute PD

Surgical treatment is usually advised for recurrent PD. On the other hand, the reported risk of redislocation after conservative treatment is extremely high [26], affecting almost 30-50% of first-time dislocators [28,46]. Recurrent PD increases the risk of chondral damage and progressive PFJ arthritis [24]. Even without recurrent dislocation, conservatively treated patients may present persisting PFJ problems. Atkin et al. reported that 58% of first-time dislocators complained of pain and restriction of strenuous activities six months after conservative treatment of the primary incidence [9].

Recent meta-analyses and systematic reviews in general favor surgical treatment; however, most authors recognize the important limitations of their studies and recommend further research on the dilemma between surgical or conservative treatment of acute PD.

Nwachukwu et al. found that conservatively treated acute dislocations had a 31% rate of recurrent dislocation rate compared to 22% in surgically treated patients. Surgical treatment may provide a better quality of life and athletic benefits [92].

Cohen et al. found a 7% rate of redislocation in the surgically treated first-time dislocators by means of MPFL reconstruction, which was significantly lower than the 30% rate of redislocation in conservatively treated patients. Moreover, the surgery group had a higher Kujala score compared to the rehabilitation group. The authors recommend early MPFL reconstruction for patients with acute primary PD [82].

Liu et al. conducted a meta-analysis to compare MPFL repair, MPFL reconstruction, and conservative treatment for patients with primary PD. The authors conclude that MPFL reconstruction is probably the best option to prevent postoperative redislocation, reduce revision rate, and improve knee function scores [19].

Other meta-analyses and systematic reviews do not provide a clear recommendation for early surgical treatment of acute PD.

The meta-analysis by Xing et al. did not find a statistical difference between conservative and surgical treatment of acute PD relative to the rate of subluxation, Kujala score, patient satisfaction, and frequency of reoperation. The Tegner activity score was significantly higher in the conservative group. However, the rate of redislocation was also significantly higher in the conservative group compared to the surgically treated group. The authors recommend further research to obtain a clear recommendation between surgical and conservative treatment of acute PD [93].

Zhang et al. compared surgical and nonsurgical treatment for primary PD in adolescents. They concluded that surgical treatment of acute PD was superior to nonsurgical treatment in the short term (up to five years postoperatively) to reduce the redislocation rate but resulted in poorer outcomes of knee function based on the Kujala and Knee Injury and Osteoarthritis Outcome Score (KOOS) scores. In the long term, surgical and conservative treatment in adolescents presents similar results [17].

Tian et al. compared conservative vs. repair of MPFL for the treatment of patients with acute primary PD. They found that only the Kujala score was higher in the surgically treated group. The two treatments did not differ significantly regarding the rate of redislocation, the percentage of excellent or good subjective opinion, the Tegner activity score, and the KOOS. MPFL repair did not produce significantly better in relation to anterior knee pain and knee activities [94].

Pagliazzi et al. found that the conservative treatment is associated with a higher redislocation rate. Furthermore, surgical treatment yields better clinical outcomes within six years after initial treatment. However, the results between surgical and conservative treatment did not differ at a longer follow-up [95].

Lee et al. concluded that surgical treatment of primary PD is not superior to conservative treatment in relation to the rate of redislocation, knee function, and clinical outcomes (Kujala score, Tegner score). However, in patients with recurrent PD, MPFL reconstruction is associated with superior clinical outcomes compared with medial soft tissue realignment surgery [96].

Longo et al. found that the rate of redislocation after surgical treatment of primary acute PD is significantly lower compared with conservative treatment (25% in the surgical group vs. 36.4% in the conservative group). In relation to the clinical outcomes, in the short term, the surgically treated patients had a significantly higher Kujala score relative to the conservatively treated patients (Kujala score 88.7 vs. 75.6 respectively). However, in the long term, both treatment groups had comparable Kujala scores (surgical treatment: 86.6, conservative treatment: 87.5) [97].

Other meta-analyses found that recurrent dislocation is less frequent after surgical treatment of primary PD; however, the authors recommended further research [98-100].

In summary, MPFL reconstruction is the first-line procedure in case surgical treatment of acute PD is decided. Additional surgery should be individualized according to the patient’s age and the severity of preexisting patellofemoral pathology.

Acute surgical treatment of primary PD is dictated by the risk of redislocation

Rupture of the medial patellar stabilizers is the most important event of traumatic lateral PD [11,12]. If the patella is anatomically reduced into the trochlea, healing of the medial stabilizers will restore the original status of the PFJ. If patellar reduction after the primary dislocation is not anatomic, which is more likely in the presence of predisposing factors of instability, the medial patellar restraints will heal at increased length, thus increasing the risk for recurrent PD. On the contrary, anatomic reduction of the patella leads to healing of the ruptured medial restraints at their original length. This is confirmed by Allen et al. who studied MPFL rupture and treatment in patients operated for multiligamentous knee injuries (MLKI) [101]. The authors found that 59% of these patients had MPFL injuries, and most were complete MPFL ruptures. Although no patient received MPFL repair or reconstruction, most patients (95%) did not have patellofemoral instability in the long term [101]. The absence of patellofemoral instability was attributed to the absence of predisposing factors for PD (especially patella alta and TD) in patients with MLKI [101].

Balcarec et al. studied 61 patients with primary PD after conservative treatment. Recurrent PD was observed in 40 patients (66%). The authors described the patellar instability score, based on six factors: age (>16 years: 0 points, ≤16 years: 1 point), bilateral instability (no: 0, yes: 1), TD (no: 0, Dejour type A-mild: 1, Dejour type BCD-severe: 2), patellar height (Insall-Salvati ≤1.2: 0, IS >1.2: 1, TT-TG distance (<16 mm: 0, ≥16 mm: 1), and patellar tilt (≤20⁰: 0, >20⁰: 1). The maximal score is seven. The risk of redislocation was 4.88 times higher in patients with a score ≥4, compared with patients with a score ≤3 [102].

Lewallen et al. studied 326 cases of primary PD in 312 patients. Most cases (n=291) were treated conservatively, and 35 cases received early surgical treatment. Of the total 326 cases, recurrent dislocation was observed in 97 cases (29.8%). The presence of patella alta, TD, and age younger than 25 years was associated with a 70.4% risk of recurrence within five years after the primary dislocation, while the presence of TD and age <25 years has a 60.2% risk of recurrence. In these patients, the authors suggest that surgical treatment of the primary PD might be preferable to conservative treatment [46].

Arendt et al. studied 145 patients with acute PD. Recurrent dislocation was observed in 61 (42%) patients. The authors found that factors, such as skeletal immaturity, MRI measurement of sulcus angle ≥154°, and Insall-Salvati ratio ≥1.3 determine the risk for recurrent PD. The probability of redislocation was 7.7%, 22.7%, 50.9%, and 78.5% with the presence of 0, 1, 2, or 3 factors, respectively [103].

Hevesi et al. followed 81 patients with primary PD, which were treated conservatively or operatively. Recurrent dislocation occurred in 38 patients (46.9%). The authors described the recurrent instability of the patella (RIP) score to predict the long-term recurrence risk after first-time dislocation, regardless of treatment. Age <25 years, skeletal maturity, TD (according to Dejour), and a TT-TG/PL (patellar length) ratio ≥0,5 were studied in patients with first-time PD. These factors received a point value of 2, 1, 1, and 1, respectively. The total sum of the point values constitutes the RIP score of the patient with primary PD. According to the RIP score, the patients were classified into low- (0-1 points), intermediate- (2-3 points), and high-risk (4-5 points) categories for recurrent PD. The 10-year risk for recurrent PD was 0.0%, 30.6%, and 79.2%, respectively. Thus, a patient with a high risk for recurrent PD could be a candidate for surgical treatment of the first PD [104].

Mochizuki et al. followed 81 patients with primary PD, which were treated conservatively. Recurrent dislocation occurred in 44 patients (54%). Thirty-four (77.3%) of these patients had free osteochondral fractures. The authors described a scoring system based on age (age <20: 2 points), history of sports injury (1 point), hemarthrosis (1 point), bony fragment (3 points), lateral shift of the patella (1 point), and TD (2 points). The maximal score was 10 points. Patients with acute PD are allocated in three groups according to the risk of recurrent PD. A score of ≤4 implies a low risk, a score of 5-7 medium risk, and ≥8 high risk for recurrent PD after conservative treatment. The authors suggest that patients with a score ≥8 should receive early surgical treatment of acute primary PD [105].

Huntington et al. conducted a meta-analysis on the factors associated with an increased risk of recurrence after a first-time PD. The overall rate of recurrence was 33.6%. Younger age, open physes, patella alta, TD, and elevated TT-TG distance were associated with an increased risk of patellar redislocation. TD was the strongest predictor of recurrence; however, the degree of dysplasia did not affect the risk for redislocation. The rate of recurrent PD was 29.6-60.2% in patients with two risk factors, while three risk factors implied a rate of recurrence of 70.4-78.5%. A seemingly controversial observation of the authors is that MPFL injury or injury pattern does not seem to be a risk factor for recurrent PD, although MPFL is the most important medial ligamentous restraint against PD. The authors explain that this is more or less expected since MPFL injury is present in almost 100% of primary PDs. Consequently, there are no patients with primary PD without an MPFL injury to serve as controls [106].

All studies show the synergistic effect of multiple risk factors on the risk of redislocation. Thus, in patients with multiple risk factors for patellar instability, early surgical treatment of the primary PD may be advisable. Furthermore, early MPFL reconstruction has been supported in the case of surgery for concomitant osteochondral injury after first-time PD [79]. Studies have shown that treating only the osteochondral injury without concomitant MPFL reconstruction in adolescents with first-time PD is associated with a high rate of redislocation [80]. Concomitant MPFL reconstruction is associated with a significant drop in the redislocation rate (10%), reduction of subsequent surgery (6.7% vs. 47.8%), and more frequent return to sports (66.7% vs. 39.1%) compared with not treating or repairing MPFL [80].

Current literature supports that the incidence of redislocation after MPFL reconstruction is lower, compared with rehabilitation; however, there is a relatively high incidence of complications such as continued apprehension and/or subluxation without dislocation [107]. Consequently, the decision between conservative or surgical treatment of primary PD is individualized, taking into account the presence of patellar instability predisposing factors. Acute primary PD in patients without or with mild underlying predisposing factors of patellar instability should be treated conservatively. However, patients with increased numbers and severity of intrinsic and extrinsic factors of patellar instability are at high risk for recurrent PD and are, therefore, strong candidates for early surgical treatment.

Isolated MPFL reconstruction

To date, an individualized approach to PD is advocated [75]. Surgical treatment of traumatic patellar instability should consider the unique anatomy and function of the PFJ. The surgical technique should not alter significantly the patients’ premorbid anatomy, but it should focus mainly on the restoration of the damaged tissue. Attempts to correct every anatomical flaw, which predisposes to patellar instability, may lead to overconstraint of the patella or to multidirectional instability.

Current evidence supports isolated MPFL reconstruction (without additional surgical treatment of other bony abnormalities) as a safe first-line procedure to restore the patellofemoral anatomy prior to the dislocation [14,79]. However, concomitant PFD, especially TD, may compromise MPFL surgery and discourage surgeons from anatomic procedures [22].

Patient characteristics that would indicate isolated MPFL reconstruction (without simultaneous bony procedures) have not been clearly defined yet [18]. The ideal candidate for isolated MPFL reconstruction could be a patient with chronic lateral patellar instability with at least two documented episodes of dislocation and confirmation of dislocation with examination under anesthesia, a TT-TG distance of less than 20 mm, a positive apprehension test up to 30° of knee flexion, the absence of patella alta, and grade A TD [108]. The graft should be stronger than the native MPFL to compensate for the uncorrected underlying PFD [109]. A double-bundle MPFL reconstruction is associated with a lower failure rate than a single-bundle reconstruction [110].

## Conclusions

The PFJ is the anatomical and functional center of the extensor mechanism of the knee. Its flawless function is crucial for walking, working, and sports participation. Acute PD is mainly a problem for younger patients in the second decade of life. In the case of competitive athletes, PD may signal the end of a career. Acute ligamentous injury of the medial patellar stabilizers after primary PD, preexisting bony pathology of the PFJ, lower limb axial and torsional deformities, and ligament laxity increase the risk of recurrent dislocation and are taken into account in treatment selection. Currently, MPFL reconstruction and restoration of axial and torsional alignment of the lower limbs are favored over non-anatomic knee-extensor-mechanism realignment procedures. Acute primary PD is usually treated conservatively. However, in the presence of factors, that predispose to patellofemoral instability, MPFL reconstruction may be the treatment of choice for acute primary PD. Improvement of core stability is an important part of the holistic management of patellar instability. Surgical treatment in skeletally immature patients is performed with caution so as not to harm the growth plates around the knee.

## Appendices

**Table 3:** Normal values for risk factors of patellofemoral instability PFJ: patellofemoral joint, MRI: magnetic resonance imaging, CT: computerized tomography, ISI: Insall-Salvati index

Normal values for risk factors of patellofemoral instability		
Factors of PFJ instability		Normal values
Patellar height	Patella alta	ISI: 0.8–1.2 [48]
	Patellotrochlear index (MRI)	Patella alta <12.5%, patella infera >50% [3]
Patellar tilt	Patellar tilt (MRI, CT)	≥20⁰ [13]
	Congruence angle	Always negative in normal knees (points medially): -6⁰ (SD=11⁰) [50]
Torsion	Femoral anteversion	24.1±17.4⁰ [57]
	Tibial external torsion (transmalleolar angle)	34.9±15.9⁰ [57]
Trochlear dysplasia	Dejour	Qualitative classification of trochlear dysplasia (A, B, C, D) [13,44]
	Lateral trochlear inclination	11–16.9° [111]
	Sulcus angle	<145⁰ [13,44,50]
	Sulcus depth, trochlear depth	The distance of the tangential to the highest points of the trochlear walls to the deepest point of the trochlear groove: 6.6 mm [112]
		The height of the lateral trochlear wall ≥5 mm [53,113]
	Trochlear facet asymmetry	Medial trochlear facet/lateral trochlear facet, normal >40%) (at 3 cm above the knee joint [2])
Valgus vector	Genu valgum	Pathological: lateral deviation from the Mikulicz line >10-15 mm [63]
		Tibiofemoral axis 6-8⁰ [63]
		In symptomatic patellar instability valgus >5⁰ may be an indication for varus osteotomy [60,88]
		Dejour: >10⁰ [13]
	TT-TG distance	Normal range: 10–15 mm [53]
		Pathologic if >15mm with maltracking, normal <20 mm [44]
	Q-angle	13⁰-15⁰, similar for men and women [52]
